# Association of glucagon-like peptide-1 receptor agonists with atrial fibrillation, cardiac arrest, and ventricular fibrillation: Casual evidence from a drug target Mendelian randomization

**DOI:** 10.1186/s13098-025-01712-w

**Published:** 2025-05-29

**Authors:** Xinyi Zhang, Nanqin Peng, Xiaoyue Zhang, Zicheng Zhu, Yan Miao, Yuting Wu, Jitao Ling, Chen Li, Wenli Gu, Jing Zhang, Abudukeremu Ayiguli, Ziheng Zheng, Peng Yu, Xiao Liu

**Affiliations:** 1 Department of Endocrinology and Metabolism, the Second Affiliated Hospital, Jiangxi Medical College, Nanchang University, Nanchang, China; 2 Department of Anesthesiology, the Second Affiliated Hospital, Jiangxi Medical College, Nanchang University, Nanchang, China; 3 Department of Pharmacy, the First Affiliated Hospital of Guangxi Medical University, Nanning, China; 4 Cardiology Division, Department of Medicine, Queen Mary Hospital, The University of Hong Kong, Hong Kong, China; 5 Department of Cardiology, Sun Yat-sen Memorial Hospital of Sun Yat-sen University, Guangzhou, China; 6 Cardiovascular & Metabolic Disorders Program, Duke-National University of Singapore Medical School, Singapore, Singapore

**Keywords:** Glucagon-like peptide-1 receptor agonists, Atrial fibrillation, Cardiac arrest, Ventricular fibrillation, Mendelian randomization

## Abstract

**Background:**

Glucagon-like peptide-1 receptor agonists (GLP-1RAs) have shown benefits for cardiorenal outcomes in patients with type 2 diabetes mellitus. Although some observational studies suggest that GLP-1RAs protect against arrhythmias, the evidence remains inconclusive.

**Methods:**

This study aimed to assess the causal relationship between GLP-1RAs and arrhythmias, including atrial fibrillation (AF), cardiac arrest, and ventricular fibrillation. We performed a two-sample Mendelian randomization (MR) analysis to examine the associations between genetically proxied GLP-1RAs and the risk of arrhythmias. Genetic instruments for GLP-1RAs were obtained from the cis-expression quantitative trait loci of the *GLP1R* gene, on the basis of data from the eQTLGen Consortium. Genome-wide association study (GWAS) data for AF were sourced from FinnGen10, whereas data for cardiac arrest and ventricular fibrillation came from the GWAS Catalog. Bayesian colocalization and multivariable Mendelian randomization (MVMR) analyses were conducted as supplementary analyses.

**Results:**

Twelve independent single nucleotide polymorphisms were identified as genetic instruments for GLP-1RAs. MR analysis indicated that genetically proxied GLP-1RAs were associated with a reduced risk of AF (odds ratio [OR] = 0.78, 95% confidence interval [CI] = 0.71–0.85, *p* = 4.45E-08, posterior probability of hypothesis 4 [PP.H4] = 0.007) and a lower risk of cardiac arrest and ventricular fibrillation (OR = 0.60, 95% CI = 0.42–0.85, *p* = 0.0039, PP.H4 = 0.018). Bayesian colocalization analysis revealed that genetically proxied GLP-1RAs did not share genetic variation with arrhythmias. MVMR analysis revealed that, after adjusting for body mass index and type 2 diabetes mellitus, genetically proxied GLP-1RAs did not have a significant effect on the risk of arrhythmias.

**Conclusions:**

Our findings suggest that genetically proxied GLP-1RAs are causally associated with a reduced risk of AF, cardiac arrest, and ventricular fibrillation. Further randomized controlled trials are needed to confirm these results.

**Supplementary Information:**

The online version contains supplementary material available at 10.1186/s13098-025-01712-w.

## Graphical abstract

**Supplementary Information:**

The online version contains supplementary material available at 10.1186/s13098-025-01712-w.

## Graphical abstract

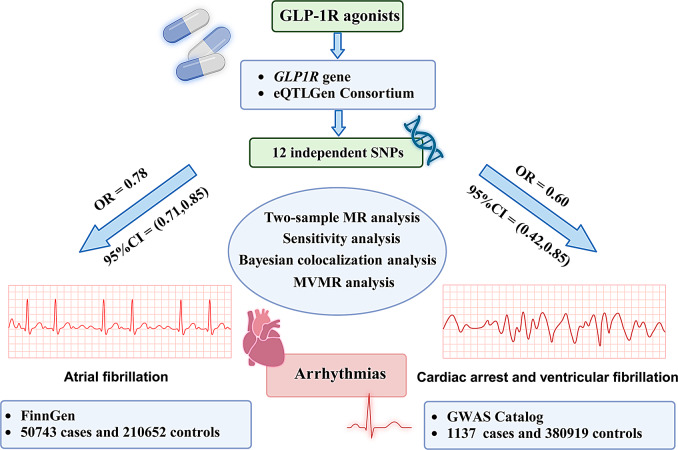

## Introduction

Cardiovascular disease is the leading cause of mortality among patients with type 2 diabetes mellitus (T2DM) and remains one of the most prevalent health concerns worldwide [1]. Individuals with T2DM face a heightened risk of developing atrial fibrillation (AF), ventricular arrhythmias, and sudden cardiac arrest [2, 3]. Moreover, prediabetes alone is a risk factor for AF, and patients with AF combined with prediabetes are at increased risk for major adverse cardiac and cerebrovascular events [4, 5]. Glucagon-like peptide-1 receptor agonists (GLP-1RAs), which are widely used for the treatment of T2DM and promote weight loss in individuals with obesity, have demonstrated significant cardiovascular benefits. Clinical trials have shown that GLP-1RAs reduce the incidence of major adverse cardiovascular events in patients with T2DM [6].

In addition to their role as hypoglycemic agents, emerging evidence suggests that GLP-1RAs may also offer protection against AF. Evidence from clinical and animal studies has demonstrated that GLP-1RAs might improve myocardial metabolism and reduce the incidence of AF [7]. A preclinical study conducted in diabetic mice revealed that liraglutide reduced AF and prevented atrial remodeling in T2DM model mice [8]. In human studies, a real-world study involving adults with diabetes in the United States found that the use of GLP-1RAs was associated with a reduced risk of AF, highlighting their potential cardiovascular benefits beyond glycemic control [9].

In addition, GLP-1RAs exert pleiotropic effects by promoting weight loss, activating GLP-1 receptors on neurons and T cells, and targeting receptors in multiple organs. These actions contribute to reduced systemic and local inflammation, improved endothelial function, and decreased oxidative stress [10]. Furthermore, GLP-1RAs have shown enhanced efficacy in improving hepatic steatosis, reducing fibrosis, and modulating inflammation, suggesting promising therapeutic potential in metabolic dysfunction-associated steatohepatitis and cardiovascular comorbidities [11].

GLP-1RAs appear to be neutral regarding the risk of ventricular arrhythmias and sudden cardiac death [3]. A meta-analysis of 5 randomized clinical trials (RCTs) found no significantly increased risk of ventricular arrhythmias or sudden cardiac death associated with GLP-1RAs in T2DM patients [12]. Similarly, another meta-analysis of 5 cardiovascular outcome trials reported no significant effect of GLP-1RAs treatment on ventricular fibrillation in T2DM patients [13]. Given these inconsistent findings, the causal relationship between GLP-1RAs and arrhythmias remains uncertain. Mendelian randomization (MR) analysis is a powerful method that examines potential causal relationships between exposure and outcome at the genetic level. Therefore, we conducted MR analysis to investigate the causal association between genetically proxied GLP-1RAs and the risk of AF, cardiac arrest, and ventricular fibrillation.

## Methods

### Study design

**Fig. 1:**
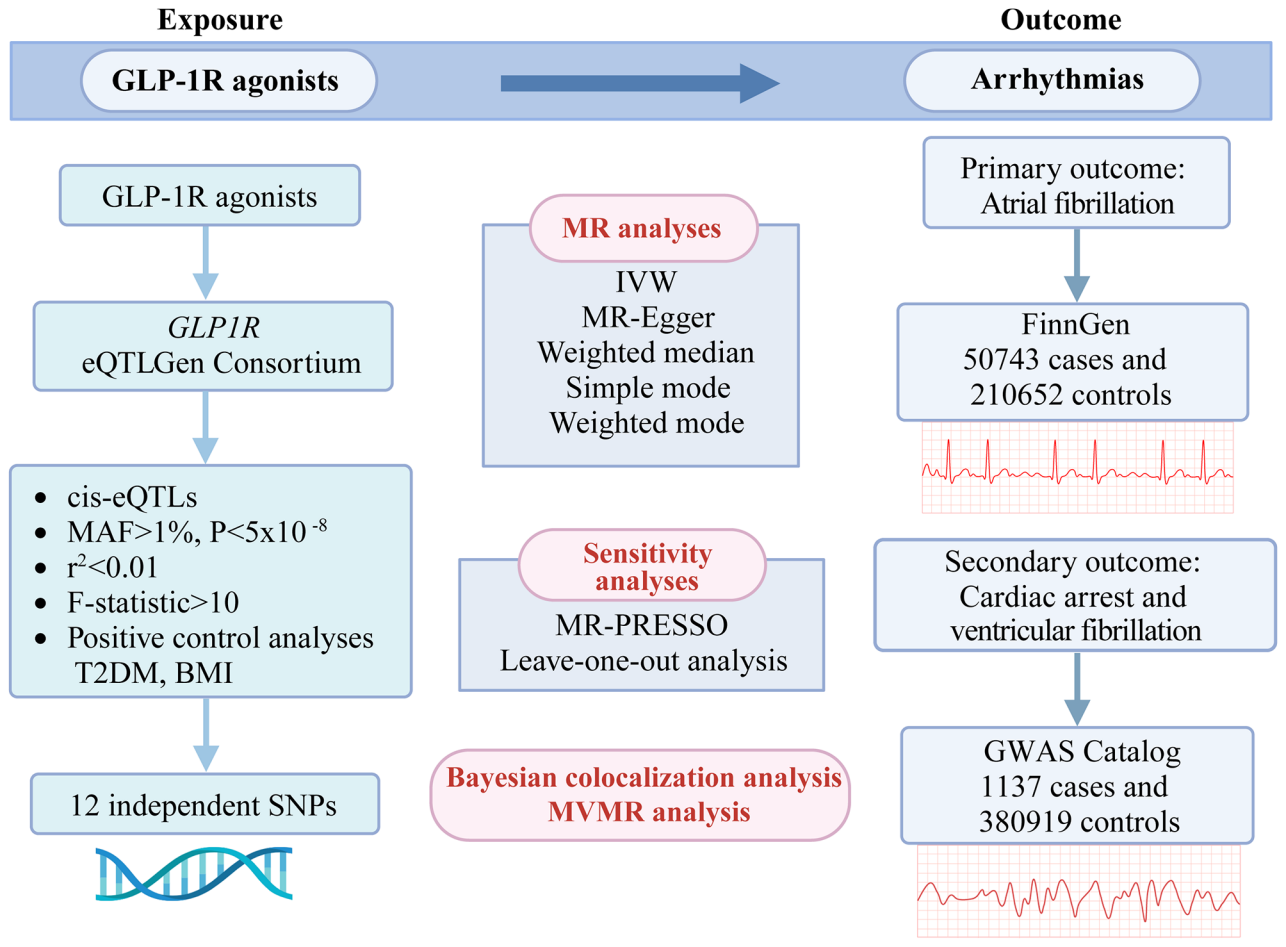
Study design overview. The flowchart of evaluating the effects of genetically proxied glucagon-like peptide-1 receptor (GLP-1R) agonists on arrhythmias. eQTL: expression quantitative trait locus; MAF: minor allele frequency; SNPs: single nucleotide polymorphisms; T2DM: type 2 diabetes mellitus; BMI: body mass index; MR: Mendelian randomization; IVW: inverse-variance weighted; MR-PRESSO: Mendelian Randomization Pleiotropy RESidual Sum and Outlier. MVMR: multivariable Mendelian randomization (Created in BioRender. Zhang, Y. (2025) https://BioRender.com/m93p747).

We conducted a two-sample MR analysis to evaluate the causal relationship between genetically proxied GLP-1RAs and arrhythmias. AF was the primary outcome, whereas cardiac arrest and ventricular fibrillation were the secondary outcomes (Fig. 1). MR analysis relies on three core assumptions: (1) genetic instruments must be strongly associated with the exposure; (2) they should not be influenced by confounding factors; and (3) they should affect outcomes only through the exposure [14]. The study was reported in accordance with the STROBE-MR (Strengthening the Reporting of Observational Studies in Epidemiology using Mendelian Randomization) guidelines [15] (Supplementary Table S1).

### Genetic instruments for GLP-1RAs

Genetic instruments for GLP-1RAs were identified on the basis of previous research [16]. We used cis-expression quantitative trait loci (cis-eQTLs) of the *GLP1R* gene as proxies for GLP-1RAs exposure. Initially, 235 single nucleotide polymorphisms (SNPs) from cis-eQTLs were obtained from the eQTLGen Consortium (Supplementary Table S2). Blood-derived expression data from 31,684 European individuals were analyzed via the eQTLGen consortium for cis- and trans-expression quantitative trait loci [17]. Second, SNPs significantly associated with *GLP1R* gene expression in blood were identified, applying a p-value threshold of 5 × 10⁻⁸ and a minor allele frequency criterion of 1%. In the next step, we performed clumping to reduce the impact of strong linkage disequilibrium, including only SNPs with weak linkage disequilibrium (r² < 0.01) (Supplementary Table S3). We further filtered out SNPs with potential pleiotropic effects on confounding factors via the LDlink tool. To validate these instruments, we conducted positive control MR analyses using T2DM and body mass index (BMI) as outcomes. The results were considered positive with a p-value < 0.05. Summary statistics for T2DM were sourced from the FinnGen12. FinnGen conducts genome-wide association study (GWAS) across a wide range of phenotypes using nationwide registries, including hospital discharge data and prescription records, and links them to genetic information [18]. The BMI data used in this study were sourced from the GIANT (Genetic Investigation of Anthropometric Traits) consortium, a large-scale international collaboration aimed at identifying genetic variants associated with BMI [19]. Lastly, F-statistics were calculated to ensure instrument strength, with values greater than 10 indicating robustness.

### Study outcomes

GWAS summary data for AF were obtained from FinnGen10. In this study, we utilized AF and atrial flutter data comprising 50,743 cases and 210,652 controls from the r10 dataset. International Classification of Diseases (ICD-10) coding is strictly followed in the diagnosis of AF (ICD codes: I48), covering paroxysmal AF, persistent AF, chronic AF, typical atrial flutter, atypical atrial flutter, and AF and atrial flutter, which are unspecified. Data for cardiac arrest and ventricular fibrillation (ICD codes: I46.0, I46.9, and I49.0) were retrieved from the GWAS Catalog database (GCST90436118), which included 1,137 cases and 380,919 controls [20]. The GWAS Catalog database, managed by the National Human Genome Research Institute and the European Bioinformatics Institute, aggregates GWAS from published research, systematically curating genetic variants associated with various traits and diseases [21].

### Mendelian randomization analysis

Genetic instruments were harmonized with exposure and outcome data to align effect alleles and remove palindromic variants (rs6458073). Two-sample MR analysis was conducted via the TwoSampleMR package (version 0.6.7) in R (version 4.3.2). We employed multiple methods, including inverse-variance weighted (IVW), MR-Egger, weighted median, simple mode, and weighted mode, considering results significant with a p-value < 0.05. The IVW method was the primary analysis method due to its robustness in the absence of horizontal pleiotropy, combining variant effects for enhanced statistical power and precision [22]. The results were visualized via scatter, funnel, and forest plots. Heterogeneity among instruments was assessed with Cochran’s Q statistics, and the MR-Egger intercept test was used to detect horizontal pleiotropy. Finally, to ensure adequate statistical power, we conducted power calculations via the online sample size and power calculator for MR developed by Stephen Burgess [23].

### Sensitivity analyses

The sensitivity analyses included the Mendelian Randomization Pleiotropy RESidual Sum and Outlier (MR-PRESSO) and leave-one-out analysis. MR-PRESSO consists of three main steps: (a) identifying horizontal pleiotropy, (b) correcting for horizontal pleiotropy by removing outliers, and (c) assessing the significance of differences in causal estimates before and after outlier removal [24]. The leave-one-out analysis was used to assess the robustness of the MR results by determining the influence of individual SNPs on the overall effect estimate.

### Bayesian colocalization analysis

Bayesian colocalization analysis was performed via the coloc.abf algorithm from the ‘coloc’ package (https://github.com/chr1swallace/coloc) to calculate the posterior probability of colocalization and ascertain whether the traits share a common genetic basis. For each locus, the Bayesian colocalization analysis evaluated five hypotheses (H0-4) regarding trait associations: H0) neither trait is associated with the locus; H1) only trait 1 is associated with the locus; H2) only trait 2 is associated with the locus; H3) both traits are associated, but with different causal variants; and H4) both traits are associated and share the same causal variant [25]. We obtained eQTL data related to the *GLP1R* gene from the eQTLGen consortium, which is cataloged in the Integrative Epidemiology Unit Open GWAS database. The eQTL data were used as trait 1 data in the colocalization analysis. The GWAS data for AF and cardiac arrest and ventricular fibrillation served as trait 2.

Following the guidelines of the “coloc” package, the SNP with the lowest p-value in the raw *GLP1R* gene eQTL data (rs9283907) was chosen as the lead SNP for the subsequent colocalization analysis. A posterior probability of hypothesis 4 (PP.H4) greater than 0.7 was generally considered strong evidence of shared causal variants ​ [26].

### Multivariable Mendelian randomization analysis

To assess whether the association between GLP1-RAs and arrhythmias is independent of BMI and T2DM, we performed multivariable Mendelian randomization (MVMR) analysis using the IVW method [27]. The instrumental variables for GLP1-RAs were selected on the basis of the criteria outlined for univariable MR analysis. The SNPs associated with BMI and T2DM at genome-wide significance (*p* < 5 × 10^− 8^) were chosen as instrumental variables, and clumping (r² < 0.01) was performed to remove linkage disequilibrium. The SNPs associated with GLP1-RAs, BMI, and T2DM were extracted as 12, 91, and 230 SNPs, respectively. These SNPs were subsequently combined across the respective exposures, and the relevant information for these SNPs was extracted from each exposure dataset. After filtering for SNPs associated with all three exposures, a total of 25 SNPs were included in the MVMR analysis.

## Results

### Primary results

We identified 12 independent SNPs as genetic instruments for the *GLP1R* gene, each with an F-statistic exceeding 40 (Table 1). Positive control analysis demonstrated that genetic instruments for the *GLP1R* gene were significantly associated with a reduced risk of T2DM (odds ratio [OR] = 0.90, 95% confidence interval [CI] = 0.86–0.96, *p* = 0.0004) and BMI (OR = 0.94, 95% CI = 0.92–0.96, *p* = 9.78E-08), validating their use as proxies for GLP-1RAs exposure (Table 2). MR analysis revealed that genetically proxied GLP-1RAs were significantly associated with a lower risk of AF (OR = 0.78, 95% CI = 0.71–0.85, *p* = 4.45E-08) (Table 3 & Supplementary Figure S1-2). Similarly, genetically proxied GLP-1RAs were linked to a reduced risk of cardiac arrest and ventricular fibrillation (OR = 0.60, 95% CI = 0.42–0.85, *p* = 0.0039) (Table 3 & Supplementary Figure S1-2). The power analysis estimated that the study had 100% power to detect associations with AF and 85.8% power to detect associations with cardiac arrest and ventricular fibrillation at a significance threshold of *p* < 0.05. No evidence of heterogeneity or horizontal pleiotropy was detected in the analysis (all *p* > 0.05) (Table 3). Funnel plots showed a symmetrical distribution of most SNPs around the central estimate, indicating the absence of significant directional pleiotropy or bias (Supplementary Figure S3).

**Table 1:** Genetic instrumental variables of GLP-1RAs for the drug target Mendelian randomization.

SNP	*P*	Effect allele	Other allele	Sample size	Eaf	Beta	Se	R2	F
rs1018437	2.02E-13	C	T	29,621	0.563498328	-0.06081376	0.008277	0.001819	53.98505
rs1678717	2.48E-13	G	A	29,623	0.566443684	-0.06062841	0.008283	0.001805	53.57585
rs1678701	3.45E-13	A	G	29,498	0.423519861	-0.06056411	0.008325	0.001791	52.92495
rs9380795	4.41E-13	A	G	29,623	0.569675674	-0.06003978	0.00829	0.001767	52.44447
rs9380787	7.07E-13	G	T	29,616	0.557232539	-0.05932389	0.008265	0.001737	51.51733
rs1738199	7.61E-13	A	G	29,505	0.565059832	-0.05946882	0.008297	0.001738	51.37531
rs9394550	1.06E-12	A	G	29,622	0.568907847	-0.05903318	0.008289	0.001709	50.71804
rs6931736	1.61E-12	A	G	29,508	0.571695478	-0.05871795	0.008312	0.001688	49.90378
rs4714193	6.26E-12	T	G	29,497	0.569478533	-0.05710797	0.008308	0.001599	47.24317
rs1678682	6.45E-12	G	A	29,508	0.565618631	-0.05700026	0.008298	0.001597	47.18271
rs1738241	7.73E-12	T	C	29,623	0.560280356	-0.056599	0.008271	0.001578	46.82896
rs114977861	2.07E-10	C	T	3243	0.012950971	0.693723719	0.109145	0.012304	40.37382

GLP-1RAs: glucagon-like peptide-1 receptor agonists; SNP: single nucleotide polymorphisms.

**Table 2:** Positive control analysis: MR estimates of the effect of genetically proxied GLP-1RAs on T2DM and BMI.

Outcome	Method	OR (95%CI)	*P*	Q statistics	*P*-heterogeneity	Egger intercept	*P* intercept
T2DM	IVW	0.90 (0.86,0.96)	0.0004	1.4829	0.9996		
	MR-Egger	0.99 (0.84,1.16)	0.9116	0.0604	1.0000	-0.0062	0.2605
	Weighted median	0.89 (0.83,0.96)	0.0022				
	Simple mode	0.89 (0.80,1.00)	0.0688				
	Weighted mode	0.89 (0.80,1.00)	0.0616				
	MR-PRESSO	0.90 (0.89,0.92)	2.07E-07	1.9559	0.9993		
BMI	IVW	0.94 (0.92,0.96)	9.78E-08	0.5469	1.0000		
	MR-Egger	0.96 (0.90,1.03)	0.2453	0.1218	1.0000	-0.0014	0.5291
	Weighted median	0.94 (0,91,0.97)	3.78E-05				
	Simple mode	0.94 (0.90,0.98)	0.0205				
	Weighted mode	0.94 (0.90,0.98)	0,0138				
	MR-PRESSO	0.94 (0,93,0.94)	6.98E-12	0.6936	1.0000		

MR: Mendelian randomization; T2DM: type 2 diabetes mellitus; BMI: body mass index; GLP-1RAs: glucagon-like peptide-1 receptor agonists; IVW: inverse-variance weighted; MR-PRESSO: Mendelian Randomization Pleiotropy RESidual Sum and Outlier; OR: odds ratio; CI: confidence interval.

**Table 3:** MR estimates of the effect of genetically proxied GLP-1RAs on atrial fibrillation and cardiac arrest and ventricular fibrillation.

Outcome	Method	OR (95%CI)	*P*	Q statistics	*P*-heterogeneity	Egger intercept	*P* intercept
**Atrial fibrillation**	IVW	0.78 (0.71,0.85)	4.45E-08	1.5541	0.9995		
	MR-Egger	0.90 (0.69,1.17)	0.4407	0.2957	1.000	-0.0097	0.2882
	Weighted median	0.76 (0.67,0.85)	5.98E-06				
	Simple mode	0.75 (0.63,0.90)	0.0105				
	Weighted mode	0.75 (0.62,0.91)	0.0156				
	MR-PRESSO	0.77 (0.75,0.80)	2.49E-09	2.0889	0.9997		
**Cardiac arrest and ventricular fibrillation**	IVW	0.60 (0.42,0.85)	0.0039	2.7257	0.9939		
	MR-Egger	0.87 (0.45,1.70)	0.6914	1.0695	0.9998	-0.0315	0.2271
	Weighted median	0.49 (0.31,0.78)	0.0027				
	Simple mode	0.47 (0.21,1.04)	0.0888				
	Weighted mode	0.83 (0.47,1.48)	0.5427				
	MR-PRESSO	0.59 (0.50,0.70)	3.91E-05	4.3376	0.9895		

MR: Mendelian randomization; GLP-1RAs: glucagon-like peptide-1 receptor agonists; OR: odds ratio; CI: confidence interval; IVW: inverse-variance weighted; MR-PRESSO: Mendelian Randomization Pleiotropy RESidual Sum and Outlier.

### Sensitivity, colocalization and multivariable Mendelian randomization analyses

The MR-PRESSO analysis identified no outliers, confirming that no SNP had a disproportionately large effect on the outcomes. The causal estimate was consistent with the IVW model (Table 3). Additionally, the leave-one-out analysis revealed that removing individual SNPs did not substantially alter the overall causal effect estimates (Supplementary Figure S4).

**Fig. 2:**
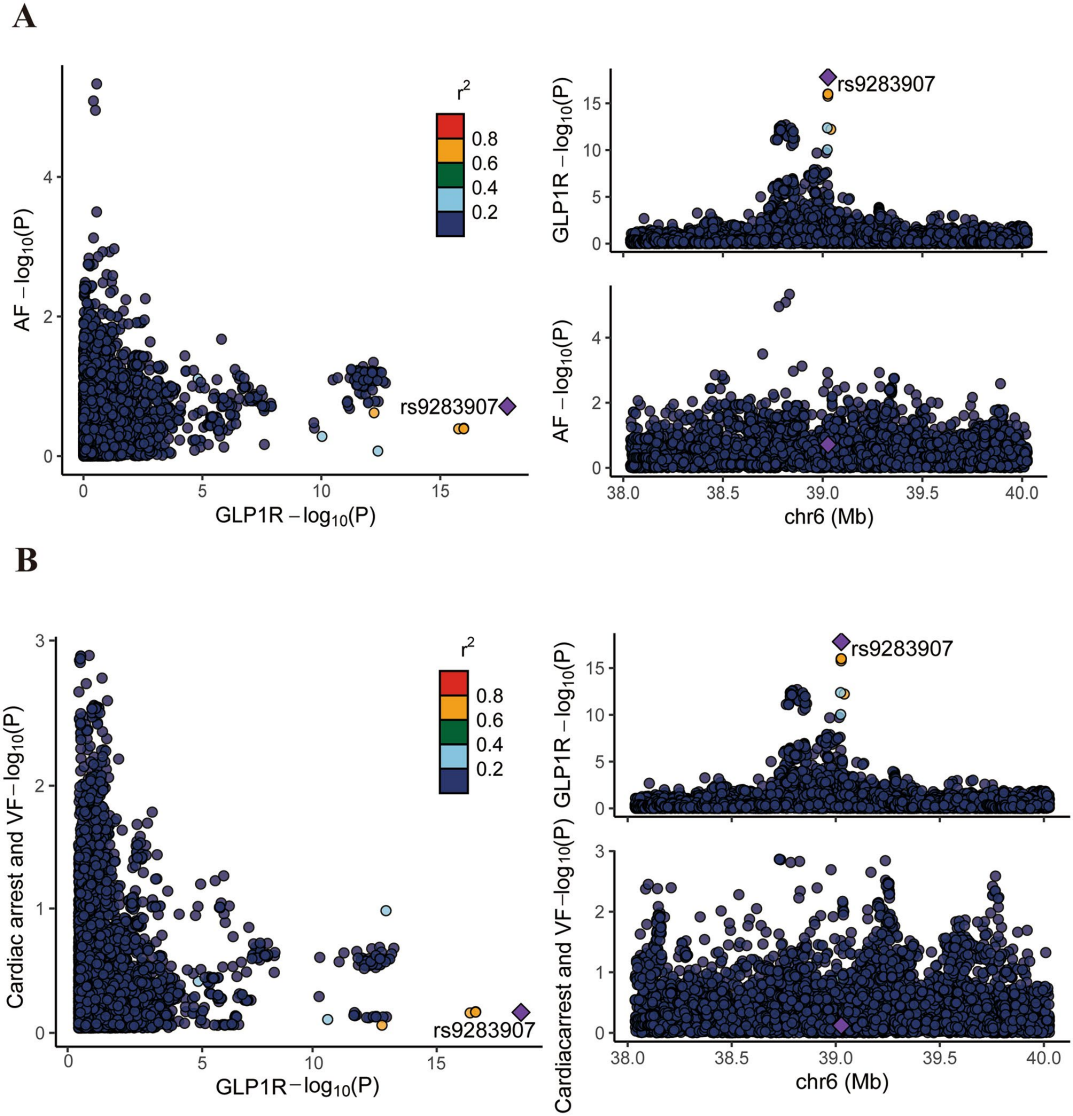
The colocalization of genetically proxied glucagon-like peptide-1 receptor agonists and arrhythmias. (**A**) Atrial fibrillation (AF); (**B**) Cardiac arrest and ventricular fibrillation (VF). Scatter plots (left) depict − log10(P) values for associations between genetic variants and *GLP1R* expression versus AF and cardiac arrest and VF, highlighting the variant rs9283907. Regional association plots (right) show the locus around rs9283907 on chromosome 6, illustrating genetic signals for both *GLP1R* expression and arrhythmias

Colocalization analysis showed no shared causal variants between genetically proxied GLP-1RAs and arrhythmias, with PP.H4 values of 0.007 for AF and 0.018 for cardiac arrest and ventricular fibrillation (Fig. 2).

MVMR analysis revealed that, after adjusting for BMI and T2DM, genetically proxied GLP-1RAs did not have a significant effect on the risk of AF (OR = 1.06, 95% CI = 0.60–1.89, *p* = 0.848) or cardiac arrest and ventricular fibrillation (OR = 1.28, 95% CI = 0.36–4.52, *p* = 0.705) (Supplementary Table S4).

## Discussion

### Major findings

Our MR analysis demonstrated that genetically proxied GLP-1RAs are causally associated with a reduced risk of AF, cardiac arrest, and ventricular fibrillation. Colocalization analysis suggested that these associations are not driven by shared genetic variants. This is the first study to establish a causal relationship between genetically proxied GLP-1RAs and arrhythmias using MR analysis.

### Comparison with previous studies

Previous *post-hoc* analyses of clinical trials and meta-analyses have investigated the effects of GLP-1RAs on AF. Several meta-analyses of RCTs have indicated that GLP-1RAs exert a neutral or potentially protective effect on AF. For example, a meta-analysis of several global multicenter clinical trials involving 12,651 patients found that semaglutide reduced AF episodes by 42% in individuals at high cardiovascular risk [28]. Another meta-analysis of 21 RCTs involving 25,957 patients with T2DM, obesity, or overweight showed that semaglutide was associated with a lower risk of AF occurrence [29]. Our findings align with these studies, reinforcing the association between GLP-1RAs and a reduced AF risk. A global retrospective cohort study utilizing data from the TriNetX global network similarly reported a significant association between GLP-1RAs treatment and a lower AF and arrhythmias risk in individuals with obesity and without T2DM [30]. However, another meta-analysis including 13 RCTs found that GLP-1RAs had a neutral effect on the occurrence of AF in populations with obesity or overweight and without diabetes [31]. Differences in study design, statistical methods, and population characteristics may explain these inconsistencies. Broader inclusion criteria in meta-analyses may dilute the observed benefits of GLP-1RAs on AF. Notably, GLP-1RAs may be more effective in patients at high risk for AF, such as those with T2DM. This hypothesis may be supported by recent findings on relationship between GLP-1RAs use and lower perioperative AF. For example, a meta-analysis demonstrated that GLP-1RAs reduced the risk of recurrent AF over a 12-month follow-up in patients undergoing AF catheter ablation, on the basis of data primarily from United States cohorts [32]. These findings suggest that the antiarrhythmic effects of GLP-1RAs may vary depending on population risk profiles. Future studies should include a broader range of high-risk populations to better define the scope of GLP-1RAs’ antiarrhythmic benefits.

The evidence linking GLP-1RAs to ventricular fibrillation and cardiac arrest is limited. A meta-analysis of 5 RCTs reported no significant impact of GLP-1RAs on ventricular arrhythmias and cardiac arrest in patients with T2DM and myocardial infarction [33]. Similarly, a nested case-control study showed no association between GLP-1RAs use and a reduced risk of out-of-hospital cardiac arrest in T2DM patients. However, stratification by sex showed a significant reduction in out-of-hospital cardiac arrest risk for women which may be related to the weight loss effect of GLP-1RAs [34]. This MR analysis identified a protective effect of genetically proxied GLP-1RAs against cardiac arrest and ventricular fibrillation, providing novel insights into the antiarrhythmic potential of GLP-1RAs. Nevertheless, further research is needed to elucidate the complex relationship between GLP-1RAs and these outcomes.

### Potential biological mechanisms

Although the colocalization analysis did not identify shared genetic variants between genetically proxied GLP-1RAs and arrhythmias, several factors may explain this finding. First, the power of colocalization analysis depends on the resolution of available GWAS datasets and the magnitude of genetic effects. Second, arrhythmias are complex polygenic traits, and the observed associations may reflect indirect pathways rather than a single shared causal variant. Additionally, GLP-1RAs exhibit pleiotropic effects on metabolic and cardiovascular pathways, which could influence arrhythmia risk through mechanisms such as glucose homeostasis, blood pressure regulation, and autonomic modulation. These alternative pathways highlight the need for further functional studies to delineate the exact mechanisms underlying the observed associations.

Previous studies have shown that GLP-1RAs improve cardiac metabolism and provide cardiovascular protection by mitigating atrial electrical and structural remodeling. GLP-1RAs have shown benefits in managing hypertension, diabetes, obesity, heart failure, atherosclerosis, and obstructive sleep apnea, which collectively lower AF risk [7]. Furthermore, GLP-1RAs enhance mitochondrial function in cardiomyocytes, reducing oxidative stress and cellular damage, thus alleviating the heart’s metabolic burden [35]. A large nationwide study of hospitalized patients has demonstrated that AF is associated with an increased risk of ventricular arrhythmias and cardiac arrest [36]. The critical role of underlying coronary artery disease (CAD) in the pathogenesis of AF and ventricular fibrillation should not be overlooked. CAD adversely affects AF progression by promoting re-entry and increasing atrial tissue excitability due to ischemia and electrical inhomogeneity. The beneficial effects of GLP-1RAs on arrhythmias may be primarily mediated through their impact on CAD [37, 38]. These mechanisms underscore the potential antiarrhythmic properties of GLP-1RAs, warranting further investigation.

### Clinical implications

Patients with T2DM and AF have higher mortality rates and cardiovascular complications than those in sinus rhythm, with thromboembolism being the most significant risk factor [2]. Therefore, the prevention of AF in diabetic patients is of clinical importance. Additionally, concerns have been raised about GLP-1RAs potentially increasing heart rate and arrhythmias [39]. Moreover, emerging evidence suggests that GLP-1RAs offer protection against AF. Our study indicates that genetically proxied GLP-1RAs have antiarrhythmic effects, which aligns with the growing body of evidence. The potential antiarrhythmic effects of GLP-1RAs provide new perspectives on the management of T2DM and its complications. The American Diabetes Association’s Standards of Care in Diabetes—2025 have recommended the use of GLP-1RAs in patients with T2DM, symptomatic heart failure with preserved ejection fraction, and obesity [40]. According to the 2024 European Society of Cardiology Guidelines for the Management of AF, hypertension, heart failure, T2DM, and obesity are recognized as risk factors contributing to both the onset and progression of AF [41]. Additionally, GLP-1RAs are recommended for patients with T2DM and chronic kidney disease, and individuals with a history of cardiovascular disease or myocardial infarction may also benefit from GLP-1RAs. Therefore, clinicians may consider prioritizing GLP-1RAs for individuals at high risk of AF, particularly those with T2DM, along with concomitant hypertension, CAD, heart failure, and obesity.

### Strengths and limitations

This is the first study to investigate the causal relationship between GLP-1RAs and arrhythmias using MR analysis, effectively addressing confounding factors and reverse causality. The use of large GWAS datasets from independent populations enhances the statistical power and robustness of the findings. However, several limitations exist. The analysis was restricted to individuals of European ancestry, limiting its generalizability to other populations. Future studies incorporating multi-ancestry genetic datasets are warranted to validate our findings and improve their external validity. The wide age range and lack of stratification by sex or AF subtypes further constrain detailed investigations. Additionally, the antiarrhythmic effects of GLP-1RAs may vary among different drug classes, a factor not examined in this study. Another limitation of this study is the lack of an explicit assessment of CAD as a mediator in the relationship between GLP-1RAs and arrhythmia risk. However, we mitigated the potential confounding effect of CAD through the use of MR analysis and by rigorously selecting genetic instruments that were strongly associated with GLP-1RAs, while excluding SNPs associated with CAD and other pleiotropic pathways. Additionally, colocalization analysis did not identify shared genetic variants, necessitating further research to uncover the underlying mechanisms involved.

Although MVMR was conducted to account for potential confounding by BMI and T2DM, several methodological limitations precluded its reliable interpretation. First, the number of shared instrumental variables was significantly reduced, leading to potential weak instrument bias and decreased statistical power. Second, GLP-1RAs instruments were derived from eQTLGen, which are inherently localized to a specific chromosomal region, whereas BMI and T2DM variants are genome-wide, limiting the availability of valid instruments for MVMR. Third, collinearity between BMI and T2DM may introduce instability into the model, further complicating interpretation. Therefore, we prioritized univariable MR analysis, which demonstrated a robust association between GLP-1RAs and arrhythmias.

## Conclusion

In summary, this MR analysis demonstrated that genetically proxied GLP-1RAs are causally associated with a reduced risk of AF, cardiac arrest, and ventricular fibrillation. These findings provide novel insights into the potential antiarrhythmic effects of GLP-1RAs. However, further large-scale RCTs are warranted to validate these results and elucidate the underlying biological mechanisms.

## Electronic supplementary material

Below is the link to the electronic supplementary material.

Supplementary Material 1

## Data Availability

No datasets were generated or analysed during the current study.

## Abbreviations

GLP-1RAs: Glucagon-like peptide-1 receptor agonists
AF: Atrial fibrillation
GWAS: Genome-wide association study
cis-eQTLs: cis-expression quantitative trait loci
OR: Odds ratio
CI: Confidence interval
T2DM: Type 2 diabetes mellitus
MR: Mendelian randomization
SNPs: Single nucleotide polymorphisms
RCTs: Randomized clinical trials
BMI: Body mass index
ICD: International Classification of Diseases
IVW: Inverse-variance weighted
MR-PRESSO: Mendelian Randomization Pleiotropy RESidual Sum and Outlier
MVMR: Multivariable Mendelian randomization
PP.H4: Posterior probability of hypothesis 4
CAD: Coronary artery disease
