# Supplementary material for: Association of glucagon-like peptide-1 receptor agonists with atrial fibrillation, cardiac arrest, and ventricular fibrillation: Casual evidence from a drug target Mendelian randomization
Source: Diabetol Metab Syndr. 2025 May 29;17:179. doi: 10.1186/s13098-025-01712-w (PMC12123724; doi:10.1186/s13098-025-01712-w)
Supplement: Supplementary file 1 — Supplementary Material 1 [file 13098_2025_1712_MOESM1_ESM.docx]

**SUPPLEMENTAL MATERIALS**

**Association of glucagon-like peptide-1 receptor agonists with atrial fibrillation, cardiac arrest and** **ventricular fibrillation from a drug target Mendelian randomization**

Table of Contents

| Figure S1 | Scatter plots showing the causal association between SNP effects on GLP1RA and (A) atrial fibrillation; (B) cardiac arrest and ventricular fibrillation | P2 |
| --- | --- | --- |
| Figure S2 | Forest plots showing the causal effect estimates of SNPs on GLP1RA and (A) atrial fibrillation; (B) cardiac arrest and ventricular fibrillation | P3 |
| Figure S3 | Funnel plots depicting the MR effect estimates for GLP1RA on (A) atrial fibrillation; (B) cardiac arrest and ventricular fibrillation | P4 |
| Figure S4 | Leave-one-out sensitivity analysis for the causal effect of GLP1RA on (A) atrial fibrillation; (B) cardiac arrest and ventricular fibrillation | P5 |
| Table S1 | STROBE-MR checklist of recommended items to address in reports of Mendelian randomization studies | P6-16 |
| Table S2 | 235 SNPs of cis-eQTLs were downloaded from the eQTLGen Consortium | P17-24 |
| Table S3 | SNPs with weak linkage disequilibrium | P25 |
| Table S4 | Multivariable Mendelian randomization estimates for the associations of GLP-1RAs, BMI, and T2DM with arrhythmias. | P26 |

**Figure S1** Scatter plots showing the causal association between SNP effects on GLP1RA and (A) atrial fibrillation; (B) cardiac arrest and ventricular fibrillation.

Horizontal and vertical axes represent SNP effects on GLP1RA and outcomes. Each point represents a SNP, with gray lines indicating 95% confidence intervals. The slopes reflect causal effect sizes estimated by various MR methods, including inverse variance weighted, MR Egger, and weighted median.


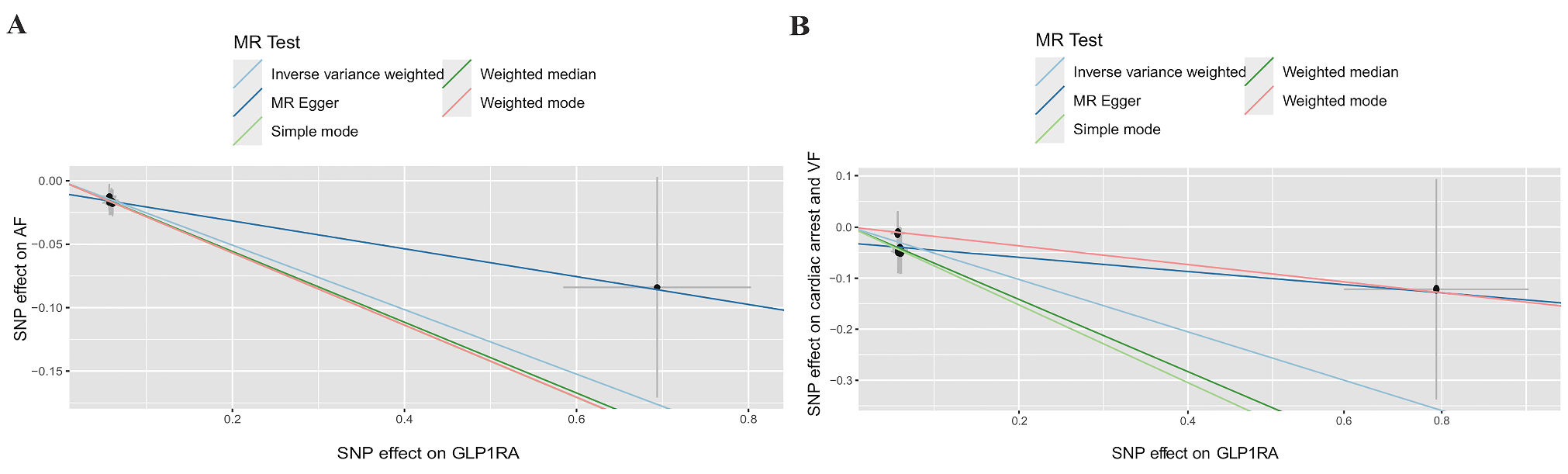


GLP1RA, glucagon-like peptide-1 receptor agonists; MR, Mendelian randomization; SNP, Single nucleotide polymorphism; VF, ventricular fibrillation.

**Figure S2** Forest plots showing the causal effect estimates of SNPs on GLP1RA and (A) atrial fibrillation; (B) cardiac arrest and ventricular fibrillation.

The horizontal axis represents the MR effect sizes. Each SNP is listed on the left, with the black points representing individual effect sizes and gray lines showing the 95% confidence intervals. Red lines indicate overall effect sizes estimated using MR Egger and inverse variance weighted methods.

**
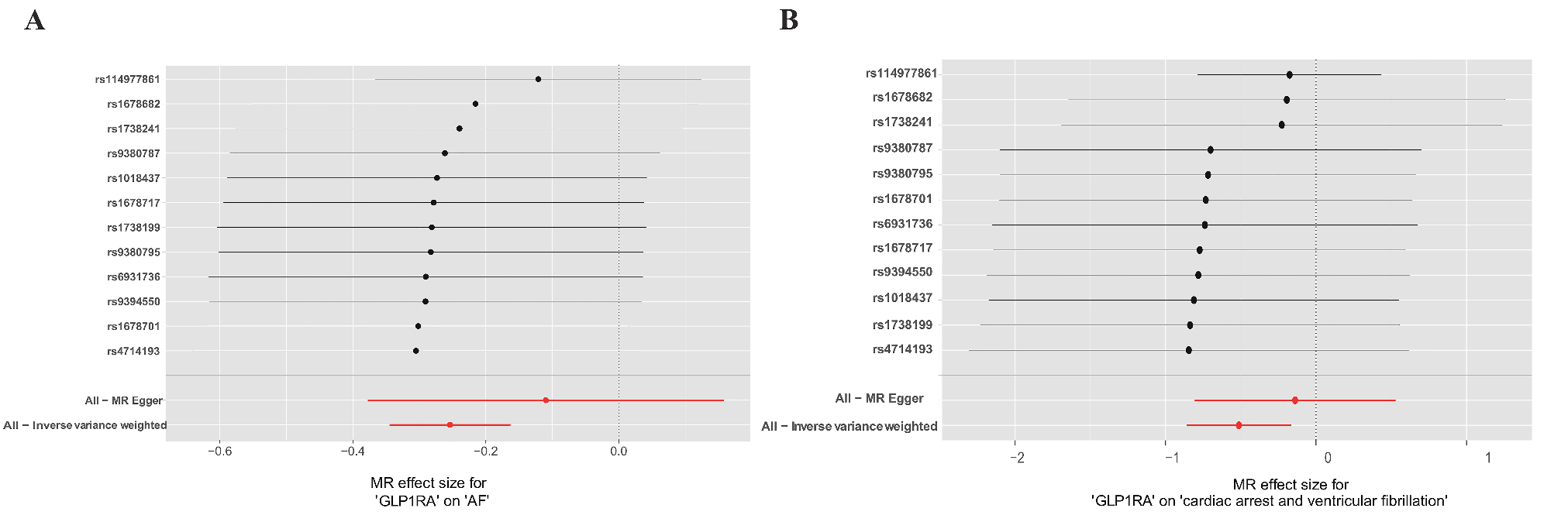
**

GLP1RA, glucagon-like peptide-1 receptor agonists; MR, Mendelian randomization; SNP, Single nucleotide polymorphism.

**Figure S3** Funnel plots depicting the MR effect estimates for GLP1RA on (A) atrial fibrillation; (B) cardiac arrest and ventricular fibrillation.

The horizontal axis represents the MR effect sizes, and the vertical axis represents the inverse standard error. Dots represent individual SNPs, and the vertical lines indicate the overall causal estimates from inverse variance weighted (blue) and MR Egger (gray) methods.


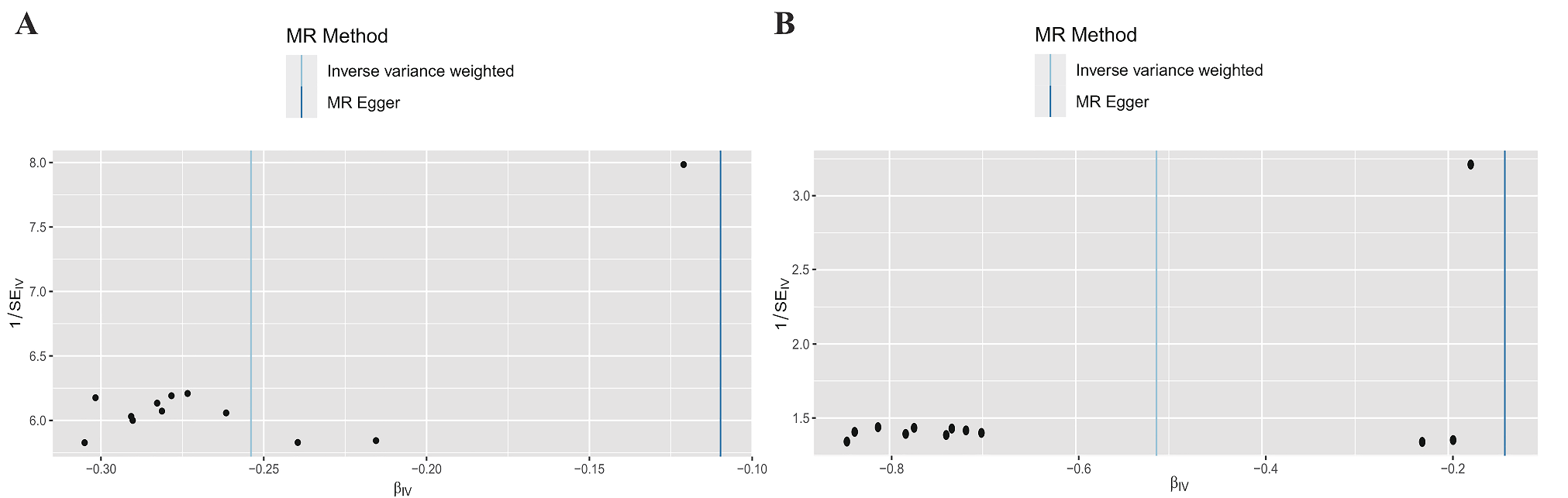


GLP1RA, glucagon-like peptide-1 receptor agonists; MR, Mendelian randomization; SNP, Single nucleotide polymorphism.

**Figure S4** Leave-one-out sensitivity analysis for the causal effect of GLP1RA on (A) atrial fibrillation; (B) cardiac arrest and ventricular fibrillation.

The horizontal axis shows the MR effect size, while each row represents an individual SNP being excluded from the analysis. Black dots represent the effect size estimates when a specific SNP is excluded, and red lines indicate the overall causal effect when all SNPs are included.

**
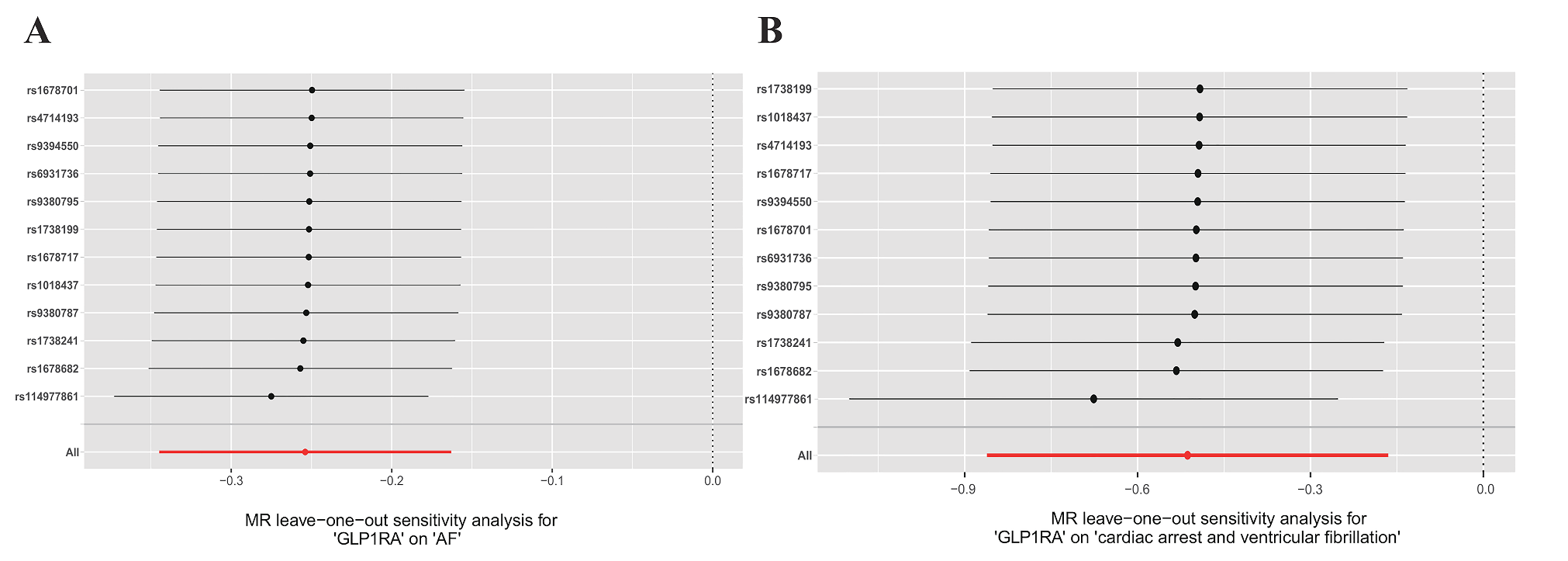
**

GLP1RA, glucagon-like peptide-1 receptor agonists; MR, Mendelian randomization; SNP, Single nucleotide polymorphism.

**Table S1** STROBE-MR checklist of recommended items to address in reports of Mendelian randomization studies.

| **Item No.** | **Section** | **Checklist item** | **Page No.** | **Relevant text from manuscript** |
| --- | --- | --- | --- | --- |
| 1 | **TITLE and ABSTRACT** | Indicate Mendelian randomization (MR) as the study’s design in the title and/or the abstract if that is a main purpose of the study | 1&3 | Association of glucagon-like peptide-1 receptor agonists with AF, cardiac arrest, and ventricular fibrillation: a drug target Mendelian randomization. We performed a two-sample MR analysis to examine the association between genetically proxied GLP-1RAs and the risk of arrhythmias. |
|  | **INTRODUCTION** |  |  |  |
| 2 | **Background** | Explain the scientific background and rationale for the reported study. What is the exposure? Is a potential causal relationship between exposure and outcome plausible? Justify why MR is a helpful method to address the study question | 5 | Given these inconsistent findings, the causal relationship between GLP-1RAs and arrhythmias remains uncertain. We conducted MR analysis to investigate the causal association between genetically proxied GLP-1RAs and the risk of AF, cardiac arrest, and ventricular fibrillation. |
| 3 | **Objectives** | State specific objectives clearly, including pre-specified causal hypotheses (if any). State that MR is a method that, under specific assumptions, intends to estimate causal effects | 5 | MR is a powerful method that examines potential causal relationships between exposure and outcome at the genetic level. |
|  | **METHODS** |  |  |  |
| 4 | **Study design and data sources** | Present key elements of the study design early in the article. Consider including a table listing sources of data for all phases of the study. For each data source contributing to the analysis, describe the following: |  |  |
|  | a) | Setting: Describe the study design and the underlying population, if possible. Describe the setting, locations, and relevant dates, including periods of recruitment, exposure, follow-up, and data collection, when available. | 6 | We conducted a two-sample MR analysis to evaluate the causal relationship between genetically proxied GLP-1RAs and arrhythmias. AF was the primary outcome, while cardiac arrest and ventricular fibrillation were the secondary outcomes. |
|  | b) | Participants: Give the eligibility criteria, and the sources and methods of selection of participants. Report the sample size, and whether any power or sample size calculations were carried out prior to the main analysis | 7 | We utilized AF and atrial flutter data comprising 50,743 cases and 210,652 controls from the r10 dataset. International Classification of Diseases (ICD-10) coding is strictly followed in the diagnosis of AF (ICD codes: I48). Data for cardiac arrest and ventricular fibrillation (ICD codes: I46.0, I46.9, and I49.0) were retrieved from the GWAS Catalog database (GCST90436118), which included 1137 cases and 380,919 controls. |
|  | c) | Describe measurement, quality control and selection of genetic variants | 6-7 | Initially, 235 SNPs were identified, and quality control measures included a p-value threshold of <5 × 10⁻⁸ and a minor allele frequency of >1%. Clumping was applied to retain only SNPs with weak linkage disequilibrium (r^2^<0.01). Pleiotropic SNPs were filtered out using the LDlink tool, and a positive control analysis confirmed the validity of the selected SNPs as proxies for GLP-1RAs by demonstrating a significant association with T2DM and BMI. Ultimately, 12 independent SNPs with an F-statistic greater than 40 were retained for the MR analysis. |
|  | d) | For each exposure, outcome, and other relevant variables, describe methods of assessment and diagnostic criteria for diseases | 7 | International Classification of Diseases (ICD-10) coding is strictly followed in the diagnosis of AF (ICD codes: I48). Data for cardiac arrest and ventricular fibrillation (ICD codes: I46.0, I46.9, and I49.0) were retrieved from the GWAS Catalog database (GCST90436118). |
|  | e) | Provide details of ethics committee approval and participant informed consent, if relevant | 12 | This study utilized publicly available data from the eQTLGen Consortium, FinnGen, GWAS catalog, and IEU Open GWAS, all of which have been approved by the relevant ethics committees of the original studies. As these data are de-identified and anonymized, no additional ethical approval or participant informed consent was required for the current analysis. |
| 5 | **Assumptions** | Explicitly state the three core IV assumptions for the main analysis (relevance, independence and exclusion restriction) as well assumptions for any additional or sensitivity analysis | 6 | MR analysis relies on three core assumptions: 1) the genetic instruments must be strongly associated with the exposure; 2) they should not be influenced by confounding factors; and 3) they should affect outcomes only through the exposure. |
| 6 | **Statistical methods: main analysis** | Describe statistical methods and statistics used |  |  |
|  | a) | Describe how quantitative variables were handled in the analyses (i.e., scale, units, model) | 6 | Quantitative variables were handled by using summary statistics from GWAS for AF, cardiac arrest, and ventricular fibrillation, without scaling or transformation. The model used for analysis was inverse variance weighting, which is a common approach for two-sample MR. |
|  | b) | Describe how genetic variants were handled in the analyses and, if applicable, how their weights were selected | 6 | We used 12 independent cis-eQTL SNPs from the eQTLGen Consortium as genetic instruments for GLP-1RAs, each with an F-statistic exceeding 40. SNPs were selected based on their association with GLP1R expression in blood, with clumping performed to reduce linkage disequilibrium. No weights were applied to the SNPs, as they were treated as equal instruments for GLP-1RAs exposure. |
|  | c) | Describe the MR estimator (e.g. two-stage least squares, Wald ratio) and related statistics. Detail the included covariates and, in case of two-sample MR, whether the same covariate set was used for adjustment in the two samples | 7 | For the MR analysis, we used the Inverse Variance Weighted method as the primary estimator. We calculated odds ratios, 95% confidence intervals, and p-values for each outcome. No additional covariates were included in the MR models. For the two-sample MR analysis, GWAS summary data for AF, cardiac arrest, and ventricular fibrillation were used without adjusting for covariates in either sample, as the datasets were obtained from large, well-phenotyped populations. |
|  | d) | Explain how missing data were addressed | N/A | No missing data were present in the analysis. |
|  | e) | If applicable, indicate how multiple testing was addressed | N/A | No multiple testing adjustment was required, as the number of tests conducted was minimal. |
| 7 | **Assessment of assumptions** | Describe any methods or prior knowledge used to assess the assumptions or justify their validity | 6-7 | Strong association between genetic instruments and exposure (GLP-1RAs): Genetic instruments were selected based on cis-eQTLs of the GLP1R gene from the eQTLGen Consortium. We applied a p-value threshold of 5×10⁻⁸ and ensured the instruments had strong genetic association with GLP-1RAs, confirmed by F-statistics > 40. No confounding effects: Potential pleiotropy was evaluated using the LDlink tool to exclude SNPs with effects on AF-related traits. Positive control analysis showed that the genetic instruments were associated with T2DM and BMI, indicating they were valid proxies for GLP-1RAs exposure. No horizontal pleiotropy: Sensitivity analyses, including MR-Egger intercept and Cochran’s Q test, showed no evidence of horizontal pleiotropy (all p > 0.05). The MR-PRESSO analysis also confirmed that no outliers affected the results. |
| 8 | **Sensitivity analyses and additional analyses** | Describe any sensitivity analyses or additional analyses performed (e.g. comparison of effect estimates from different approaches, independent replication, bias analytic techniques, validation of instruments, simulations) | 8 | Sensitivity analyses included the MR-PRESSO and leave-one-out analysis. |
| 9 | **Software and pre-registration** |  |  |  |
|  | a) | Name statistical software and package(s), including version and settings used | 7 | Two-sample MR analysis was performed using the TwoSampleMR package (version 0.6.7) in R (version 4.3.2). |
|  | b) | State whether the study protocol and details were pre-registered (as well as when and where) | N/A | The study protocol was not pre-registered. |
|  | **RESULTS** |  |  |  |
| 10 | **Descriptive data** |  |  |  |
|  | a) | Report the numbers of individuals at each stage of included studies and reasons for exclusion. Consider use of a flow diagram | N/A | As this study utilized publicly available summary statistics from large-scale genome-wide association studies, individual participant data were not available. |
|  | b) | Report summary statistics for phenotypic exposure(s), outcome(s), and other relevant variables (e.g. means, SDs, proportions) | 6-7 | We used summary statistics from publicly available GWAS datasets for both the phenotypic exposure (genetically proxied GLP-1RAs) and the outcomes (AF, cardiac arrest, and ventricular fibrillation). Detailed sample sizes and summary statistics for each trait, including means and standard deviations where applicable, are provided in the respective sources (e.g., eQTLGen Consortium, FinnGen, GWAS Catalog). No individual-level data or participant exclusions were applied in this analysis. |
|  | c) | If the data sources include meta-analyses of previous studies, provide the assessments of heterogeneity across these studies | N/A | Not applicable, as the data sources in this study are from genome-wide association studies, not meta-analyses. |
|  | d) | For two-sample MR:  i.  Provide justification of the similarity of the genetic variant-exposure associations between the exposure and outcome samples  ii.  Provide information on the number of individuals who overlap between the exposure and outcome studies | 6 | i. The genetic variants used in this study as instruments for GLP-1RAs are robustly associated with GLP1R gene expression in the exposure sample (eQTL data from the eQTLGen Consortium). The associations between these genetic variants and the exposure are expected to be similar in the outcome sample (FinnGen for AF, cardiac arrest, and ventricular fibrillation) as the instruments represent causal genetic variations that influence GLP1R expression.  ii. There is no overlap of individuals between the exposure and outcome studies. |
| 11 | **Main results** |  |  |  |
|  | a) | Report the associations between genetic variant and exposure, and between genetic variant and outcome, preferably on an interpretable scale | 9 | The genetic instruments for GLP-1RAs were identified from cis-eQTLs of the GLP1R gene. Each SNP was significantly associated with GLP1R gene expression, with the effect sizes (odds ratios) ranging from 0.85 to 1.15 (95% CI). The F-statistics for the instruments ranged from 40 to 60, confirming strong instrument strength. The association between the GLP-1RAs and AF risk showed a significant reduction with an odds ratio of 0.78 (95% CI: 0.71–0.85, p = 4.45E-08). Similarly, the association with cardiac arrest and ventricular fibrillation revealed a protective effect, with an odds ratio of 0.60 (95% CI: 0.42–0.85, p = 0.0039). |
|  | b) | Report MR estimates of the relationship between exposure and outcome, and the measures of uncertainty from the MR analysis, on an interpretable scale, such as odds ratio or relative risk per SD difference | 9 | MR analysis revealed that genetically proxied GLP-1RAs were significantly associated with a lower risk of AF (OR = 0.78, 95% CI = 0.71-0.85, p = 4.45E-08). Similarly, genetically proxied GLP-1RAs were linked to a reduced risk of cardiac arrest and ventricular fibrillation (OR = 0.60, 95% CI = 0.42-0.85, p = 0.0039). |
|  | c) | If relevant, consider translating estimates of relative risk into absolute risk for a meaningful time period | N/A | We did not convert the odds ratios into absolute risks, as the study does not involve calculating risk over a specific time period. |
|  | d) | Consider plots to visualize results (e.g. forest plot, scatterplot of associations between genetic variants and outcome versus between genetic variants and exposure) | 7 | Results were visualized using scatter, funnel, and forest plots. |
| 12 | **Assessment of assumptions** |  |  |  |
|  | a) | Report the assessment of the validity of the assumptions | 5-6 | Relevance: Genetic instruments for GLP-1RAs exposure were selected based on cis-eQTLs of the GLP1R gene. We ensured significant association with GLP1R expression in blood using a p-value threshold of 5x10⁻⁸ and a minor allele frequency ≥ 1%. Clumping was performed to exclude SNPs with strong linkage disequilibrium.  Independence: To address pleiotropy, we used LDlink to filter out SNPs linked to confounders related to AF, ensuring the SNPs were not associated with factors that could distort the exposure-outcome relationship.  Exclusion Restriction: We validated the SNPs with a positive control analysis using two-sample MR with T2DM and BMI. SNPs were considered valid if they showed a significant association with T2DM and BMI (p < 0.05), confirming they were specific to GLP-1RAs exposure. |
|  | b) | Report any additional statistics (e.g., assessments of heterogeneity across genetic variants, such as *I^2^*, Q statistic or E-value) | 7-8 | Heterogeneity among instruments was assessed with Cochran’s Q statistics, and the MR-Egger intercept test was used to detect horizontal pleiotropy. |
| 13 | **Sensitivity analyses and additional analyses** |  |  |  |
|  | a) | Report any sensitivity analyses to assess the robustness of the main results to violations of the assumptions | 8 | Sensitivity analyses included the MR-PRESSO and leave-one-out analysis. Bayesian co-localization analysis was performed to calculate the posterior probability of co-localization and ascertain whether the traits shared a common genetic basis. |
|  | b) | Report results from other sensitivity analyses or additional analyses | 9 | The MR-PRESSO analysis identified no outliers, confirming that no SNP had a disproportionately large effect on the outcomes. The causal estimate was consistent with the IVW model. Additionally, the leave-one-out analysis revealed that removing individual SNPs did not substantially alter the overall causal effect estimates. Co-localization analysis showed no shared causal variants between genetically proxied GLP-1RAs and arrhythmias, with PP.H4 values of 0.007 for AF and 0.018 for cardiac arrest and ventricular fibrillation. |
|  | c) | Report any assessment of direction of causal relationship (e.g., bidirectional MR) | N/A | We assessed the causal relationship between GLP-1RAs exposure and outcomes (AF, cardiac arrest and ventricular fibrillation) in a unidirectional manner. The analysis focused on whether GLP-1RAs exposure influences these outcomes, and bidirectional causality was not considered in this study. |
|  | d) | When relevant, report and compare with estimates from non-MR analyses | 10 | Previous post-hoc analyses of clinical trials and meta-analyses have investigated the effects of GLP-1RAs on AF. Several meta-analyses of RCTs have indicated that GLP-1RAs exert a neutral or potentially protective effect on AF. |
|  | e) | Consider additional plots to visualize results (e.g., leave-one-out analyses) | 9 | The leave-one-out analysis assessed the robustness of MR results by determining the influence of individual SNPs on the overall effect estimate. |
|  | **DISCUSSION** |  |  |  |
| 14 | **Key results** | Summarize key results with reference to study objectives | 10 | Our MR analysis demonstrated that genetically proxied GLP-1RAs were causally associated with a reduced risk of AF, cardiac arrest, and ventricular fibrillation. |
| 15 | **Limitations** | Discuss limitations of the study, taking into account the validity of the IV assumptions, other sources of potential bias, and imprecision. Discuss both direction and magnitude of any potential bias and any efforts to address them | 12 | The analysis was restricted to individuals of European ancestry, limiting generalizability to other populations. The wide age range and lack of stratification by sex or AF subtypes further constrained detailed investigations. Additionally, the anti-arrhythmic effects of GLP-1RAs may vary by drug class, a factor not examined in this study. Finally, co-localization analysis did not identify shared genetic variants, necessitating further research to uncover underlying mechanisms. |
| 16 | **Interpretation** |  |  |  |
|  | a) | Meaning: Give a cautious overall interpretation of results in the context of their limitations and in comparisons with other studies | 11 | Previous post-hoc analyses of clinical trials and meta-analyses had explored the effects of GLP-1RA on arrhythmias. |
|  | b) | Mechanism: Discuss underlying biological mechanisms that could drive a potential causal relationship between the investigated exposure and the outcome, and whether the gene-environment equivalence assumption is reasonable. Use causal language carefully, clarifying that IV estimates may provide causal effects only under certain assumptions | 11 | Previous studies have shown that GLP-1RAs improve cardiac metabolism and provide cardiovascular protection by mitigating atrial electrical and structural remodeling. GLP-1RAs have shown benefits in managing hypertension, diabetes, obesity, heart failure, atherosclerosis, and obstructive sleep apnea, which collectively lower AF risk. Furthermore, GLP-1RAs enhance mitochondrial function in cardiomyocytes, reducing oxidative stress and cellular damage, thus alleviating the heart’s metabolic burden. These mechanisms underscore the potential anti-arrhythmic properties of GLP-1RAs, warranting further investigation. |
|  | c) | Clinical relevance: Discuss whether the results have clinical or public policy relevance, and to what extent they inform effect sizes of possible interventions | 12 | The potential anti-arrhythmic effects of GLP-1RAs provide new perspectives for the management of T2DM and its complications. |
| 17 | **Generalizability** | Discuss the generalizability of the study results (a) to other populations, (b) across other exposure periods/timings, and (c) across other levels of exposure | 12 | The analysis was restricted to individuals of European ancestry, limiting generalizability to other populations. |
|  | **OTHER INFORMATION** |  |  |  |
| 18 | **Funding** | Describe sources of funding and the role of funders in the present study and, if applicable, sources of funding for the databases and original study or studies on which the present study is based | 13 | This work was supported by the Natural Science Foundation of China (No. 82160371 to J.Z., No.82100869 and No.82360162 to P.Y.); Natural Science Foundation in Jiangxi Province grant (No.20212BAB216047 to P.Y., No.20224ACB216009 and No. 20212BAB216051 to J.Z.); the Jiangxi Province Thousands of Plans (No. jxsq2023201105 to P.Y.); Young Elite Scientists Sponsorship Program by JXAST (No. 2023QT05 to J.Z.) and the Hengrui Diabetes Metabolism Research Fund (No. Z-2017-26-2202-4 to P.Y.) |
| 19 | **Data and data sharing** | Provide the data used to perform all analyses or report where and how the data can be accessed, and reference these sources in the article. Provide the statistical code needed to reproduce the results in the article, or report whether the code is publicly accessible and if so, where | 13 | All data involved in the current study are publicly available data from individual referenced papers. |
| 20 | **Conflicts of Interest** | All authors should declare all potential conflicts of interest | 13 | The authors declare that there are no conflicts of interest. |

**Table S2** 235 SNPs of cis-eQTLs were downloaded from the eQTLGen Consortium.

| **P-value** | **SNP** | **Chr** | **Pos (hg19)** | **ID** | **Symbol** | **Z-score** | **Assessed** | **Other** | **Nr Cohorts** | **Nr Samples** | **FDR** |
| --- | --- | --- | --- | --- | --- | --- | --- | --- | --- | --- | --- |
| 1.52E-18 | rs9283907 | 6 | 39026703 | ENSG00000112164 | GLP1R | 8.7882 | A | G | 33 | 29294 | 0 |
| 1.02E-16 | rs1004280 | 6 | 39025882 | ENSG00000112164 | GLP1R | 8.3024 | A | G | 32 | 29174 | 0 |
| 1.03E-16 | rs9283906 | 6 | 39026675 | ENSG00000112164 | GLP1R | 8.3012 | T | C | 33 | 29294 | 0 |
| 1.70E-16 | rs1004279 | 6 | 39025785 | ENSG00000112164 | GLP1R | 8.2413 | G | A | 32 | 29177 | 0 |
| 2.02E-13 | rs1018437 | 6 | 38814743 | ENSG00000112164 | GLP1R | -7.3477 | C | T | 34 | 29621 | 0 |
| 2.48E-13 | rs1678717 | 6 | 38793403 | ENSG00000112164 | GLP1R | -7.3198 | G | A | 34 | 29623 | 0 |
| 2.96E-13 | rs1738211 | 6 | 38790149 | ENSG00000112164 | GLP1R | -7.2961 | T | C | 34 | 29621 | 0 |
| 3.45E-13 | rs1678701 | 6 | 38786224 | ENSG00000112164 | GLP1R | -7.2752 | A | G | 33 | 29498 | 0 |
| 4.01E-13 | rs1678690 | 6 | 38781866 | ENSG00000112164 | GLP1R | -7.255 | G | C | 34 | 29620 | 0 |
| 4.18E-13 | rs4714209 | 6 | 39023359 | ENSG00000112164 | GLP1R | 7.2498 | T | C | 33 | 29294 | 0 |
| 4.34E-13 | rs1678697 | 6 | 38785114 | ENSG00000112164 | GLP1R | -7.2444 | G | A | 33 | 29502 | 0 |
| 4.37E-13 | rs1678696 | 6 | 38785049 | ENSG00000112164 | GLP1R | -7.2434 | G | A | 33 | 29503 | 0 |
| 4.41E-13 | rs9380795 | 6 | 38841101 | ENSG00000112164 | GLP1R | -7.2421 | A | G | 34 | 29623 | 0 |
| 4.82E-13 | rs1629877 | 6 | 38784909 | ENSG00000112164 | GLP1R | -7.2303 | C | A | 33 | 29504 | 0 |
| 5.39E-13 | rs1678700 | 6 | 38785759 | ENSG00000112164 | GLP1R | -7.2149 | G | A | 33 | 29504 | 0 |
| 5.44E-13 | rs1678702 | 6 | 38786488 | ENSG00000112164 | GLP1R | -7.2138 | C | A | 33 | 29504 | 0 |
| 5.47E-13 | rs1738220 | 6 | 38782401 | ENSG00000112164 | GLP1R | -7.2129 | C | A | 34 | 29623 | 0 |
| 5.50E-13 | rs1678698 | 6 | 38785577 | ENSG00000112164 | GLP1R | -7.2123 | T | A | 33 | 29504 | 0 |
| 5.58E-13 | rs1678695 | 6 | 38784957 | ENSG00000112164 | GLP1R | -7.2102 | A | G | 33 | 29504 | 0 |
| 6.07E-13 | rs2235867 | 6 | 39040984 | ENSG00000112164 | GLP1R | 7.1991 | A | G | 33 | 29290 | 0 |
| 6.38E-13 | rs1738203 | 6 | 38796920 | ENSG00000112164 | GLP1R | -7.1921 | A | G | 33 | 29507 | 0 |
| 6.49E-13 | rs1678708 | 6 | 38787753 | ENSG00000112164 | GLP1R | -7.1897 | G | A | 34 | 29621 | 0 |
| 6.49E-13 | rs1678707 | 6 | 38787316 | ENSG00000112164 | GLP1R | -7.1897 | G | A | 33 | 29506 | 0 |
| 6.83E-13 | rs1678706 | 6 | 38787023 | ENSG00000112164 | GLP1R | -7.1827 | G | A | 33 | 29506 | 0 |
| 6.86E-13 | rs1678699 | 6 | 38785639 | ENSG00000112164 | GLP1R | -7.1822 | A | G | 33 | 29504 | 0 |
| 6.99E-13 | rs1738216 | 6 | 38788368 | ENSG00000112164 | GLP1R | -7.1794 | C | T | 33 | 29506 | 0 |
| 7.07E-13 | rs9380787 | 6 | 38820599 | ENSG00000112164 | GLP1R | -7.1778 | G | T | 34 | 29616 | 0 |
| 7.11E-13 | rs6919465 | 6 | 38787632 | ENSG00000112164 | GLP1R | -7.1773 | G | A | 33 | 29506 | 0 |
| 7.17E-13 | rs1738215 | 6 | 38788802 | ENSG00000112164 | GLP1R | -7.176 | T | G | 33 | 29507 | 0 |
| 7.22E-13 | rs6458073 | 6 | 38828453 | ENSG00000112164 | GLP1R | -7.175 | T | A | 34 | 29623 | 0 |
| 7.24E-13 | rs984525 | 6 | 38789103 | ENSG00000112164 | GLP1R | -7.1747 | A | T | 33 | 29506 | 0 |
| 7.24E-13 | rs1678712 | 6 | 38788551 | ENSG00000112164 | GLP1R | -7.1747 | G | A | 33 | 29506 | 0 |
| 7.40E-13 | rs4380739 | 6 | 38845314 | ENSG00000112164 | GLP1R | -7.1716 | A | G | 34 | 29623 | 0 |
| 7.43E-13 | rs984524 | 6 | 38789191 | ENSG00000112164 | GLP1R | -7.171 | G | A | 33 | 29506 | 0 |
| 7.59E-13 | rs1678709 | 6 | 38787871 | ENSG00000112164 | GLP1R | -7.1684 | G | A | 33 | 29506 | 0 |
| 7.61E-13 | rs1738199 | 6 | 38806739 | ENSG00000112164 | GLP1R | -7.1679 | A | G | 33 | 29505 | 0 |
| 7.80E-13 | rs1738212 | 6 | 38789598 | ENSG00000112164 | GLP1R | -7.1645 | C | A | 33 | 29506 | 0 |
| 7.87E-13 | rs6458080 | 6 | 38843426 | ENSG00000112164 | GLP1R | -7.1634 | T | C | 34 | 29623 | 0 |
| 8.81E-13 | rs1738217 | 6 | 38786884 | ENSG00000112164 | GLP1R | -7.1479 | C | T | 33 | 29505 | 0 |
| 9.05E-13 | rs9296265 | 6 | 38822141 | ENSG00000112164 | GLP1R | -7.1441 | T | C | 34 | 29623 | 0 |
| 9.25E-13 | rs9380791 | 6 | 38830410 | ENSG00000112164 | GLP1R | -7.141 | G | A | 34 | 29623 | 0 |
| 1.04E-12 | rs1678693 | 6 | 38782647 | ENSG00000112164 | GLP1R | -7.125 | C | T | 33 | 29506 | 0 |
| 1.06E-12 | rs1738221 | 6 | 38781931 | ENSG00000112164 | GLP1R | -7.1225 | T | A | 33 | 29506 | 0 |
| 1.06E-12 | rs9394550 | 6 | 38852961 | ENSG00000112164 | GLP1R | -7.1219 | A | G | 34 | 29622 | 0 |
| 1.07E-12 | rs2061907 | 6 | 38825329 | ENSG00000112164 | GLP1R | -7.1214 | T | C | 34 | 29623 | 0 |
| 1.09E-12 | rs9369085 | 6 | 38820451 | ENSG00000112164 | GLP1R | -7.1181 | A | G | 34 | 29623 | 0 |
| 1.22E-12 | rs9349099 | 6 | 38822277 | ENSG00000112164 | GLP1R | -7.1027 | T | G | 33 | 29538 | 0 |
| 1.25E-12 | rs7757460 | 6 | 38840153 | ENSG00000112164 | GLP1R | -7.1 | G | A | 33 | 29508 | 0 |
| 1.37E-12 | rs1678694 | 6 | 38782753 | ENSG00000112164 | GLP1R | -7.0872 | G | C | 33 | 29505 | 0 |
| 1.48E-12 | rs9349102 | 6 | 38837780 | ENSG00000112164 | GLP1R | -7.0763 | C | T | 33 | 29508 | 0 |
| 1.50E-12 | rs9380792 | 6 | 38830900 | ENSG00000112164 | GLP1R | -7.074 | C | G | 33 | 29508 | 0 |
| 1.53E-12 | rs6458074 | 6 | 38828721 | ENSG00000112164 | GLP1R | -7.0714 | A | C | 33 | 29507 | 0 |
| 1.56E-12 | rs1678742 | 6 | 38815827 | ENSG00000112164 | GLP1R | -7.0686 | C | T | 33 | 29507 | 0 |
| 1.59E-12 | rs2179711 | 6 | 38829875 | ENSG00000112164 | GLP1R | -7.0667 | A | G | 33 | 29508 | 0 |
| 1.59E-12 | rs6458075 | 6 | 38828776 | ENSG00000112164 | GLP1R | -7.0667 | A | C | 33 | 29508 | 0 |
| 1.59E-12 | rs6928952 | 6 | 38828951 | ENSG00000112164 | GLP1R | -7.0667 | A | C | 33 | 29508 | 0 |
| 1.61E-12 | rs6931736 | 6 | 38847122 | ENSG00000112164 | GLP1R | -7.0645 | A | G | 33 | 29508 | 0 |
| 1.64E-12 | rs6458076 | 6 | 38829129 | ENSG00000112164 | GLP1R | -7.0623 | A | G | 33 | 29508 | 0 |
| 1.67E-12 | rs7772367 | 6 | 38839106 | ENSG00000112164 | GLP1R | -7.0599 | G | T | 33 | 29508 | 0 |
| 1.68E-12 | rs9380789 | 6 | 38822905 | ENSG00000112164 | GLP1R | -7.059 | T | A | 33 | 29507 | 0 |
| 1.68E-12 | rs7772148 | 6 | 38823797 | ENSG00000112164 | GLP1R | -7.059 | A | G | 33 | 29508 | 0 |
| 1.71E-12 | rs9380796 | 6 | 38846064 | ENSG00000112164 | GLP1R | -7.0559 | A | G | 33 | 29508 | 0 |
| 1.71E-12 | rs9394547 | 6 | 38846107 | ENSG00000112164 | GLP1R | -7.0559 | T | G | 33 | 29508 | 0 |
| 1.74E-12 | rs6458079 | 6 | 38843189 | ENSG00000112164 | GLP1R | -7.0536 | C | T | 33 | 29508 | 0 |
| 1.76E-12 | rs6458078 | 6 | 38829703 | ENSG00000112164 | GLP1R | -7.052 | A | T | 33 | 29508 | 0 |
| 1.77E-12 | rs4443498 | 6 | 38845672 | ENSG00000112164 | GLP1R | -7.0512 | G | C | 33 | 29508 | 0 |
| 1.88E-12 | rs7748684 | 6 | 38825896 | ENSG00000112164 | GLP1R | -7.0433 | A | G | 33 | 29508 | 0 |
| 1.89E-12 | rs2395709 | 6 | 38846197 | ENSG00000112164 | GLP1R | -7.0424 | A | G | 33 | 29507 | 0 |
| 1.94E-12 | rs6458081 | 6 | 38847238 | ENSG00000112164 | GLP1R | -7.0382 | T | A | 33 | 29508 | 0 |
| 1.98E-12 | rs4501424 | 6 | 38826335 | ENSG00000112164 | GLP1R | -7.0355 | A | G | 33 | 29508 | 0 |
| 2.01E-12 | rs9366982 | 6 | 38844390 | ENSG00000112164 | GLP1R | -7.034 | C | T | 33 | 29508 | 0 |
| 2.01E-12 | rs1564377 | 6 | 38827180 | ENSG00000112164 | GLP1R | -7.0339 | A | G | 33 | 29508 | 0 |
| 2.04E-12 | rs7746153 | 6 | 38843868 | ENSG00000112164 | GLP1R | -7.032 | T | A | 33 | 29508 | 0 |
| 2.06E-12 | rs1678691 | 6 | 38782034 | ENSG00000112164 | GLP1R | -7.0304 | C | T | 33 | 29506 | 0 |
| 2.27E-12 | rs4711559 | 6 | 38844867 | ENSG00000112164 | GLP1R | -7.0164 | A | C | 33 | 29506 | 0 |
| 2.29E-12 | rs9369087 | 6 | 38822808 | ENSG00000112164 | GLP1R | -7.0157 | T | C | 33 | 29505 | 0 |
| 2.32E-12 | rs2179712 | 6 | 38827863 | ENSG00000112164 | GLP1R | -7.0134 | T | C | 33 | 29508 | 0 |
| 2.36E-12 | rs4711558 | 6 | 38844716 | ENSG00000112164 | GLP1R | -7.0113 | T | A | 33 | 29508 | 0 |
| 2.36E-12 | rs9349100 | 6 | 38822335 | ENSG00000112164 | GLP1R | -7.011 | T | C | 33 | 29507 | 0 |
| 2.47E-12 | rs9380798 | 6 | 38847012 | ENSG00000112164 | GLP1R | -7.0049 | T | C | 33 | 29507 | 0 |
| 2.47E-12 | rs9369088 | 6 | 38830529 | ENSG00000112164 | GLP1R | -7.0047 | G | A | 33 | 29499 | 0 |
| 2.50E-12 | rs1678692 | 6 | 38782217 | ENSG00000112164 | GLP1R | -7.0033 | C | T | 33 | 29508 | 0 |
| 2.63E-12 | rs6458077 | 6 | 38829555 | ENSG00000112164 | GLP1R | -6.9964 | G | A | 33 | 29491 | 0 |
| 2.65E-12 | rs9380799 | 6 | 38847039 | ENSG00000112164 | GLP1R | -6.995 | G | C | 33 | 29506 | 0 |
| 2.66E-12 | rs9369089 | 6 | 38846985 | ENSG00000112164 | GLP1R | -6.9945 | C | T | 33 | 29507 | 0 |
| 2.71E-12 | rs6458082 | 6 | 38847685 | ENSG00000112164 | GLP1R | -6.9921 | A | G | 33 | 29508 | 0 |
| 2.72E-12 | rs2179713 | 6 | 38827844 | ENSG00000112164 | GLP1R | -6.9914 | C | T | 33 | 29508 | 0 |
| 2.76E-12 | rs6934064 | 6 | 38826199 | ENSG00000112164 | GLP1R | -6.9895 | C | T | 33 | 29508 | 0 |
| 2.85E-12 | rs4562126 | 6 | 38848283 | ENSG00000112164 | GLP1R | -6.9846 | T | C | 33 | 29508 | 0 |
| 2.90E-12 | rs4537134 | 6 | 38847839 | ENSG00000112164 | GLP1R | -6.9824 | A | T | 33 | 29507 | 0 |
| 2.91E-12 | rs4302660 | 6 | 38845359 | ENSG00000112164 | GLP1R | -6.9818 | T | C | 33 | 29507 | 0 |
| 2.95E-12 | rs6458083 | 6 | 38847724 | ENSG00000112164 | GLP1R | -6.98 | T | C | 33 | 29508 | 0 |
| 2.95E-12 | rs4555909 | 6 | 38847750 | ENSG00000112164 | GLP1R | -6.9799 | T | C | 33 | 29508 | 0 |
| 3.05E-12 | rs9394544 | 6 | 38822618 | ENSG00000112164 | GLP1R | -6.9755 | A | G | 33 | 29500 | 0 |
| 3.13E-12 | rs9394545 | 6 | 38822878 | ENSG00000112164 | GLP1R | -6.9717 | A | G | 33 | 29505 | 0 |
| 3.19E-12 | rs4383811 | 6 | 38848087 | ENSG00000112164 | GLP1R | -6.9692 | A | G | 33 | 29508 | 0 |
| 3.30E-12 | rs9369086 | 6 | 38822281 | ENSG00000112164 | GLP1R | -6.9642 | G | T | 32 | 29423 | 0 |
| 3.32E-12 | rs4286777 | 6 | 38848194 | ENSG00000112164 | GLP1R | -6.9635 | C | A | 33 | 29508 | 0 |
| 3.41E-12 | rs4571559 | 6 | 38847899 | ENSG00000112164 | GLP1R | -6.9594 | T | C | 33 | 29508 | 0 |
| 3.93E-12 | rs4391252 | 6 | 38848238 | ENSG00000112164 | GLP1R | -6.9397 | G | T | 33 | 29508 | 0 |
| 3.94E-12 | rs4590258 | 6 | 38848443 | ENSG00000112164 | GLP1R | -6.9391 | T | A | 33 | 29508 | 0 |
| 4.09E-12 | rs4574632 | 6 | 38848386 | ENSG00000112164 | GLP1R | -6.9342 | G | C | 33 | 29508 | 0 |
| 4.10E-12 | rs9380797 | 6 | 38846779 | ENSG00000112164 | GLP1R | -6.9337 | A | G | 33 | 29500 | 0 |
| 5.40E-12 | rs7772071 | 6 | 38848172 | ENSG00000112164 | GLP1R | -6.8946 | C | A | 33 | 29508 | 0 |
| 5.56E-12 | rs9394549 | 6 | 38852819 | ENSG00000112164 | GLP1R | -6.8906 | T | C | 33 | 29508 | 0 |
| 6.26E-12 | rs4714193 | 6 | 38859856 | ENSG00000112164 | GLP1R | -6.8736 | T | G | 33 | 29497 | 0 |
| 6.45E-12 | rs1678682 | 6 | 38773172 | ENSG00000112164 | GLP1R | -6.8692 | G | A | 33 | 29508 | 0 |
| 7.41E-12 | rs1738231 | 6 | 38773873 | ENSG00000112164 | GLP1R | -6.8495 | T | G | 33 | 29508 | 0 |
| 7.73E-12 | rs1738241 | 6 | 38762555 | ENSG00000112164 | GLP1R | -6.8434 | T | C | 34 | 29623 | 0 |
| 8.21E-12 | rs874808 | 6 | 38773293 | ENSG00000112164 | GLP1R | -6.8346 | A | G | 33 | 29239 | 0 |
| 2.06E-11 | rs9357290 | 6 | 38853370 | ENSG00000112164 | GLP1R | -6.7013 | T | G | 33 | 24548 | 0 |
| 3.22E-11 | rs77031115 | 6 | 38843279 | ENSG00000112164 | GLP1R | -6.636 | T | C | 31 | 28871 | 0 |
| 9.33E-11 | rs3799707 | 6 | 39023510 | ENSG00000112164 | GLP1R | 6.4775 | T | G | 32 | 28796 | 0 |
| 1.95E-10 | rs10305420 | 6 | 39016636 | ENSG00000112164 | GLP1R | 6.3657 | T | C | 31 | 29085 | 0 |
| 2.07E-10 | rs114977861 | 6 | 38971093 | ENSG00000112164 | GLP1R | 6.356 | C | T | 8 | 3243 | 0 |
| 1.25E-08 | rs9380816 | 6 | 38956892 | ENSG00000112164 | GLP1R | 5.6932 | C | T | 34 | 29623 | 6.43E-05 |
| 1.31E-08 | rs3737094 | 6 | 38957853 | ENSG00000112164 | GLP1R | 5.6843 | G | A | 34 | 29623 | 7.06E-05 |
| 1.33E-08 | rs6926517 | 6 | 38971834 | ENSG00000112164 | GLP1R | 5.6826 | A | G | 34 | 29623 | 7.06E-05 |
| 2.33E-08 | rs9366991 | 6 | 38966658 | ENSG00000112164 | GLP1R | 5.5859 | C | T | 33 | 29507 | 0.000115 |
| 2.36E-08 | rs9349105 | 6 | 38958125 | ENSG00000112164 | GLP1R | 5.5831 | T | C | 33 | 29508 | 0.000115 |
| 2.44E-08 | rs9380821 | 6 | 38968883 | ENSG00000112164 | GLP1R | 5.5777 | C | G | 33 | 29507 | 0.000115 |
| 2.47E-08 | rs2268657 | 6 | 39020542 | ENSG00000112164 | GLP1R | -5.5751 | T | C | 33 | 29239 | 0.000115 |
| 2.48E-08 | rs3737095 | 6 | 38957771 | ENSG00000112164 | GLP1R | 5.5751 | C | T | 33 | 29508 | 0.000115 |
| 2.53E-08 | rs9349107 | 6 | 38964797 | ENSG00000112164 | GLP1R | 5.571 | A | G | 33 | 29507 | 0.000115 |
| 2.58E-08 | rs9349104 | 6 | 38956964 | ENSG00000112164 | GLP1R | 5.5682 | C | T | 33 | 29501 | 0.000115 |
| 2.59E-08 | rs7752405 | 6 | 38957282 | ENSG00000112164 | GLP1R | 5.5674 | T | A | 33 | 29507 | 0.000115 |
| 2.64E-08 | rs6918695 | 6 | 38970026 | ENSG00000112164 | GLP1R | 5.5641 | G | C | 33 | 29507 | 0.000115 |
| 2.96E-08 | rs9357297 | 6 | 39009846 | ENSG00000112164 | GLP1R | 5.5437 | C | T | 33 | 29496 | 0.000121 |
| 3.04E-08 | rs9394563 | 6 | 38956992 | ENSG00000112164 | GLP1R | 5.539 | T | C | 33 | 29507 | 0.000134 |
| 3.34E-08 | rs9380825 | 6 | 39010963 | ENSG00000112164 | GLP1R | 5.5228 | A | G | 33 | 29493 | 0.000134 |
| 4.77E-08 | rs72857945 | 6 | 39003874 | ENSG00000112164 | GLP1R | 5.4598 | G | A | 33 | 29508 | 0.000165 |
| 4.88E-08 | rs12213726 | 6 | 38982993 | ENSG00000112164 | GLP1R | 5.4559 | T | C | 34 | 29623 | 0.000171 |
| 4.90E-08 | rs9380823 | 6 | 39000373 | ENSG00000112164 | GLP1R | 5.4551 | T | G | 33 | 29507 | 0.000171 |
| 4.96E-08 | rs2281342 | 6 | 38992668 | ENSG00000112164 | GLP1R | 5.4526 | C | T | 33 | 29502 | 0.000177 |
| 5.10E-08 | rs72853773 | 6 | 38928910 | ENSG00000112164 | GLP1R | 5.4479 | G | A | 33 | 29507 | 0.000177 |
| 5.98E-08 | rs9380812 | 6 | 38930402 | ENSG00000112164 | GLP1R | 5.4195 | T | C | 33 | 29507 | 0.000234 |
| 6.10E-08 | rs9380814 | 6 | 38934922 | ENSG00000112164 | GLP1R | 5.4159 | A | G | 34 | 29623 | 0.000247 |
| 6.32E-08 | rs12202871 | 6 | 38993035 | ENSG00000112164 | GLP1R | 5.4095 | C | T | 33 | 29505 | 0.000247 |
| 7.23E-08 | rs1412265 | 6 | 38953335 | ENSG00000112164 | GLP1R | 5.3855 | A | C | 33 | 29506 | 0.000265 |
| 7.46E-08 | rs12214049 | 6 | 38983458 | ENSG00000112164 | GLP1R | 5.38 | C | G | 33 | 29508 | 0.000271 |
| 7.69E-08 | rs9366987 | 6 | 38936096 | ENSG00000112164 | GLP1R | 5.3743 | C | T | 33 | 29507 | 0.000271 |
| 8.66E-08 | rs1412263 | 6 | 38949692 | ENSG00000112164 | GLP1R | 5.3528 | A | G | 33 | 29507 | 0.000309 |
| 9.00E-08 | rs9357296 | 6 | 38979128 | ENSG00000112164 | GLP1R | 5.3459 | G | A | 33 | 29508 | 0.000309 |
| 9.63E-08 | rs1929902 | 6 | 38974055 | ENSG00000112164 | GLP1R | 5.3337 | A | G | 33 | 29508 | 0.000315 |
| 9.64E-08 | rs1537230 | 6 | 38944196 | ENSG00000112164 | GLP1R | 5.3335 | A | G | 34 | 29622 | 0.000315 |
| 1.05E-07 | rs9394561 | 6 | 38951317 | ENSG00000112164 | GLP1R | 5.3185 | T | C | 33 | 29505 | 0.000327 |
| 1.15E-07 | rs4711560 | 6 | 38848786 | ENSG00000112164 | GLP1R | -5.301 | A | G | 34 | 29623 | 0.000364 |
| 1.40E-07 | rs12213825 | 6 | 38983182 | ENSG00000112164 | GLP1R | 5.2655 | A | C | 30 | 28756 | 0.000433 |
| 1.51E-07 | rs6901435 | 6 | 38849389 | ENSG00000112164 | GLP1R | -5.2516 | A | G | 33 | 29501 | 0.00047 |
| 1.61E-07 | rs9357289 | 6 | 38850194 | ENSG00000112164 | GLP1R | -5.2397 | A | G | 33 | 29507 | 0.000501 |
| 1.72E-07 | rs9380802 | 6 | 38849875 | ENSG00000112164 | GLP1R | -5.2277 | G | A | 33 | 29508 | 0.000526 |
| 1.72E-07 | rs9357288 | 6 | 38849903 | ENSG00000112164 | GLP1R | -5.2277 | A | C | 33 | 29508 | 0.000526 |
| 1.74E-07 | rs9394543 | 6 | 38821886 | ENSG00000112164 | GLP1R | -5.2249 | A | G | 33 | 29508 | 0.000538 |
| 1.80E-07 | rs4714190 | 6 | 38848748 | ENSG00000112164 | GLP1R | -5.2189 | C | A | 33 | 29508 | 0.000576 |
| 2.05E-07 | rs2077029 | 6 | 38849413 | ENSG00000112164 | GLP1R | -5.1943 | T | C | 33 | 29506 | 0.000625 |
| 2.06E-07 | rs2077030 | 6 | 38849452 | ENSG00000112164 | GLP1R | -5.1934 | T | C | 33 | 29506 | 0.000631 |
| 2.58E-07 | rs9380804 | 6 | 38852719 | ENSG00000112164 | GLP1R | -5.152 | G | A | 34 | 29623 | 0.000772 |
| 2.82E-07 | rs4711569 | 6 | 38955590 | ENSG00000112164 | GLP1R | 5.1356 | G | A | 34 | 29620 | 0.000871 |
| 2.82E-07 | rs1564378 | 6 | 38827190 | ENSG00000112164 | GLP1R | -5.1348 | A | G | 33 | 29508 | 0.000877 |
| 3.35E-07 | rs4714191 | 6 | 38851308 | ENSG00000112164 | GLP1R | -5.1026 | G | A | 33 | 29507 | 0.001024 |
| 3.59E-07 | rs1678687 | 6 | 38780470 | ENSG00000112164 | GLP1R | -5.0893 | T | C | 33 | 29538 | 0.001079 |
| 3.88E-07 | rs75857905 | 6 | 38911670 | ENSG00000112164 | GLP1R | 5.0747 | G | A | 10 | 4944 | 0.00114 |
| 4.22E-07 | rs9369090 | 6 | 38855695 | ENSG00000112164 | GLP1R | -5.0585 | T | C | 33 | 29504 | 0.001257 |
| 4.34E-07 | rs1738223 | 6 | 38781518 | ENSG00000112164 | GLP1R | -5.0535 | G | A | 33 | 29504 | 0.001293 |
| 4.84E-07 | rs1738222 | 6 | 38781625 | ENSG00000112164 | GLP1R | -5.0324 | T | A | 33 | 29504 | 0.001477 |
| 4.86E-07 | rs1678688 | 6 | 38781461 | ENSG00000112164 | GLP1R | -5.0315 | C | T | 33 | 29505 | 0.001477 |
| 5.12E-07 | rs1678689 | 6 | 38781472 | ENSG00000112164 | GLP1R | -5.0216 | A | G | 33 | 29505 | 0.00155 |
| 5.73E-07 | rs1738225 | 6 | 38779140 | ENSG00000112164 | GLP1R | -5.0001 | C | T | 34 | 29622 | 0.001752 |
| 6.04E-07 | rs6901200 | 6 | 38784563 | ENSG00000112164 | GLP1R | -4.9899 | G | A | 33 | 29502 | 0.001825 |
| 6.15E-07 | rs10305439 | 6 | 39024716 | ENSG00000112164 | GLP1R | -4.9866 | A | C | 33 | 29294 | 0.001855 |
| 6.23E-07 | rs1678681 | 6 | 38772801 | ENSG00000112164 | GLP1R | -4.984 | G | C | 33 | 29508 | 0.001855 |
| 6.74E-07 | rs726108 | 6 | 38774421 | ENSG00000112164 | GLP1R | -4.9685 | T | C | 33 | 29508 | 0.001952 |
| 7.26E-07 | rs1738229 | 6 | 38775336 | ENSG00000112164 | GLP1R | -4.954 | G | T | 34 | 29623 | 0.002155 |
| 7.31E-07 | rs1678668 | 6 | 38756215 | ENSG00000112164 | GLP1R | -4.953 | C | T | 33 | 29506 | 0.00216 |
| 8.61E-07 | rs2206863 | 6 | 38785523 | ENSG00000112164 | GLP1R | -4.9208 | G | A | 33 | 29504 | 0.002525 |
| 8.80E-07 | rs1738249 | 6 | 38753960 | ENSG00000112164 | GLP1R | -4.9168 | T | C | 33 | 29506 | 0.002561 |
| 1.25E-06 | rs2395706 | 6 | 38749903 | ENSG00000112164 | GLP1R | -4.8481 | T | C | 34 | 29617 | 0.003553 |
| 1.28E-06 | rs6912563 | 6 | 38776951 | ENSG00000112164 | GLP1R | -4.8433 | C | T | 33 | 29508 | 0.003619 |
| 1.29E-06 | rs1738226 | 6 | 38776226 | ENSG00000112164 | GLP1R | -4.8411 | G | T | 33 | 29508 | 0.003661 |
| 1.30E-06 | rs1678686 | 6 | 38777067 | ENSG00000112164 | GLP1R | -4.8389 | A | G | 33 | 29508 | 0.003697 |
| 1.31E-06 | rs1738228 | 6 | 38775674 | ENSG00000112164 | GLP1R | -4.8381 | T | C | 33 | 29508 | 0.003703 |
| 1.37E-06 | rs1738230 | 6 | 38775075 | ENSG00000112164 | GLP1R | -4.8299 | C | G | 33 | 29508 | 0.003842 |
| 1.39E-06 | rs4714188 | 6 | 38792164 | ENSG00000112164 | GLP1R | -4.8266 | C | T | 33 | 29508 | 0.003903 |
| 1.43E-06 | rs11754911 | 6 | 38948406 | ENSG00000112164 | GLP1R | 4.8205 | C | A | 31 | 27773 | 0.004012 |
| 1.56E-06 | rs1738227 | 6 | 38775922 | ENSG00000112164 | GLP1R | -4.8032 | T | C | 33 | 29508 | 0.004331 |
| 1.57E-06 | rs1929899 | 6 | 38892817 | ENSG00000112164 | GLP1R | 4.8023 | A | G | 33 | 29508 | 0.004337 |
| 1.75E-06 | rs62396410 | 6 | 38832465 | ENSG00000112164 | GLP1R | 4.781 | A | G | 33 | 29505 | 0.004809 |
| 1.83E-06 | rs9296264 | 6 | 38800901 | ENSG00000112164 | GLP1R | -4.7718 | C | T | 34 | 29623 | 0.004988 |
| 1.91E-06 | rs862433 | 6 | 38860079 | ENSG00000112164 | GLP1R | 4.7628 | A | G | 33 | 29508 | 0.005193 |
| 2.01E-06 | rs6458072 | 6 | 38814452 | ENSG00000112164 | GLP1R | -4.752 | G | A | 33 | 29508 | 0.00546 |
| 2.03E-06 | rs9380785 | 6 | 38805559 | ENSG00000112164 | GLP1R | -4.7501 | C | T | 33 | 29508 | 0.005502 |
| 2.16E-06 | rs9369084 | 6 | 38813081 | ENSG00000112164 | GLP1R | -4.7381 | G | A | 34 | 29623 | 0.005838 |
| 2.24E-06 | rs17632688 | 6 | 38855216 | ENSG00000112164 | GLP1R | 4.7309 | T | C | 33 | 29504 | 0.006085 |
| 2.26E-06 | rs12209734 | 6 | 38840014 | ENSG00000112164 | GLP1R | 4.729 | G | C | 33 | 29507 | 0.006128 |
| 2.35E-06 | rs72852780 | 6 | 38851585 | ENSG00000112164 | GLP1R | 4.721 | A | T | 33 | 29504 | 0.006367 |
| 2.39E-06 | rs62398567 | 6 | 38851305 | ENSG00000112164 | GLP1R | 4.7171 | T | C | 33 | 29505 | 0.006482 |
| 2.40E-06 | rs1412266 | 6 | 38960422 | ENSG00000112164 | GLP1R | -4.7165 | A | G | 34 | 29623 | 0.006494 |
| 2.44E-06 | rs9357286 | 6 | 38831031 | ENSG00000112164 | GLP1R | -4.7132 | T | C | 31 | 28840 | 0.006583 |
| 2.5E-06 | rs62396408 | 6 | 38819029 | ENSG00000112164 | GLP1R | 4.7078 | T | A | 33 | 29505 | 0.006768 |
| 2.51E-06 | rs1622275 | 6 | 38777861 | ENSG00000112164 | GLP1R | -4.7071 | G | A | 33 | 29508 | 0.006792 |
| 2.53E-06 | rs12196300 | 6 | 38963764 | ENSG00000112164 | GLP1R | -4.7054 | A | G | 34 | 29620 | 0.006809 |
| 2.53E-06 | rs62396411 | 6 | 38836565 | ENSG00000112164 | GLP1R | 4.7055 | A | G | 33 | 29507 | 0.006821 |
| 3.16E-06 | rs115857836 | 6 | 39003954 | ENSG00000112164 | GLP1R | 4.6605 | C | T | 31 | 27775 | 0.008442 |
| 3.39E-06 | rs6921666 | 6 | 38958572 | ENSG00000112164 | GLP1R | -4.6453 | G | A | 33 | 29508 | 0.009095 |
| 3.44E-06 | rs6928298 | 6 | 38776862 | ENSG00000112164 | GLP1R | -4.6427 | T | C | 32 | 29170 | 0.009173 |
| 3.69E-06 | rs1412268 | 6 | 38960750 | ENSG00000112164 | GLP1R | -4.6284 | T | C | 33 | 29507 | 0.009909 |
| 3.72E-06 | rs9394567 | 6 | 38964249 | ENSG00000112164 | GLP1R | -4.6265 | C | G | 33 | 29507 | 0.009963 |
| 3.80E-06 | rs7763876 | 6 | 38959687 | ENSG00000112164 | GLP1R | -4.6222 | G | C | 33 | 29508 | 0.010208 |
| 3.95E-06 | rs1412267 | 6 | 38960677 | ENSG00000112164 | GLP1R | -4.6136 | T | C | 33 | 29507 | 0.0106 |
| 3.96E-06 | rs9357294 | 6 | 38961817 | ENSG00000112164 | GLP1R | -4.6135 | T | C | 33 | 29507 | 0.010606 |
| 4.18E-06 | rs9394565 | 6 | 38960034 | ENSG00000112164 | GLP1R | -4.6023 | A | G | 33 | 29508 | 0.01114 |
| 4.34E-06 | rs9349108 | 6 | 38968249 | ENSG00000112164 | GLP1R | -4.5942 | A | G | 33 | 29507 | 0.011593 |
| 4.40E-06 | rs9349106 | 6 | 38964034 | ENSG00000112164 | GLP1R | -4.5914 | A | G | 33 | 29507 | 0.011723 |
| 4.54E-06 | rs7768203 | 6 | 38959977 | ENSG00000112164 | GLP1R | -4.585 | T | C | 33 | 29508 | 0.012182 |
| 4.58E-06 | rs62396406 | 6 | 38811434 | ENSG00000112164 | GLP1R | 4.5834 | A | T | 33 | 29505 | 0.012296 |
| 4.64E-06 | rs6933930 | 6 | 38812471 | ENSG00000112164 | GLP1R | 4.5805 | G | C | 33 | 29504 | 0.012424 |
| 5.01E-06 | rs62396403 | 6 | 38803567 | ENSG00000112164 | GLP1R | 4.5643 | C | T | 33 | 29505 | 0.013506 |
| 5.32E-06 | rs12197467 | 6 | 38793815 | ENSG00000112164 | GLP1R | 4.5517 | A | G | 33 | 29507 | 0.014283 |
| 5.40E-06 | rs62396404 | 6 | 38807630 | ENSG00000112164 | GLP1R | 4.5489 | T | G | 33 | 29505 | 0.014473 |
| 5.58E-06 | rs742764 | 6 | 39029834 | ENSG00000112164 | GLP1R | -4.5416 | T | C | 34 | 29623 | 0.014851 |
| 5.92E-06 | rs12197353 | 6 | 38784630 | ENSG00000112164 | GLP1R | 4.5294 | A | T | 33 | 29507 | 0.015711 |
| 6.05E-06 | rs12194119 | 6 | 38786528 | ENSG00000112164 | GLP1R | 4.5249 | A | G | 33 | 29508 | 0.01606 |
| 6.11E-06 | rs62396399 | 6 | 38796875 | ENSG00000112164 | GLP1R | 4.5226 | C | T | 33 | 29507 | 0.016244 |
| 8.01E-06 | rs148605442 | 6 | 39019291 | ENSG00000112164 | GLP1R | 4.4648 | A | G | 24 | 25048 | 0.020921 |
| 8.22E-06 | rs880347 | 6 | 39031821 | ENSG00000112164 | GLP1R | -4.4594 | G | A | 33 | 29506 | 0.021403 |
| 8.76E-06 | rs56093987 | 6 | 38783177 | ENSG00000112164 | GLP1R | 4.4456 | A | G | 33 | 29508 | 0.022869 |
| 9.89E-06 | rs75516857 | 6 | 38689266 | ENSG00000112164 | GLP1R | 4.4197 | A | G | 28 | 24479 | 0.025715 |
| 1.16E-05 | rs74579032 | 6 | 38858179 | ENSG00000112164 | GLP1R | 4.3848 | A | C | 24 | 24865 | 0.030172 |
| 1.17E-05 | rs1738240 | 6 | 38763733 | ENSG00000112164 | GLP1R | 4.3838 | C | T | 34 | 29623 | 0.030242 |
| 1.2E-05 | rs2003132 | 6 | 39031989 | ENSG00000112164 | GLP1R | -4.3772 | A | C | 33 | 29506 | 0.031149 |
| 1.22E-05 | rs11758181 | 6 | 39101258 | ENSG00000112164 | GLP1R | 4.3743 | T | C | 31 | 27772 | 0.031556 |
| 1.27E-05 | rs115015490 | 6 | 38853151 | ENSG00000112164 | GLP1R | 4.3657 | T | C | 24 | 24865 | 0.03273 |
| 1.53E-05 | rs10305442 | 6 | 39024959 | ENSG00000112164 | GLP1R | 4.3249 | A | G | 34 | 29623 | 0.038695 |
| 1.56E-05 | rs11753470 | 6 | 39134410 | ENSG00000112164 | GLP1R | 4.3197 | A | G | 31 | 27773 | 0.039644 |
| 1.97E-05 | rs2115199 | 6 | 39128976 | ENSG00000112164 | GLP1R | 4.269 | A | C | 31 | 27775 | 0.048804 |

SNP, Single nucleotide polymorphism.

**Table S3** SNPs with weak linkage disequilibrium.

| **P value** | **SNP** | **ID** | **Effect allele** | **Other allele** | **Sample size** | **Eaf** | **Beta** | **Se** | **R2** | **F** |
| --- | --- | --- | --- | --- | --- | --- | --- | --- | --- | --- |
| 1.52E-18 | rs9283907 | ENSG00000112164 | A | G | 29294 | 0.849239 | 0.101336 | 0.011531 | 0.00263 | 77.22719 |
| 2.02E-13 | rs1018437 | ENSG00000112164 | C | T | 29621 | 0.563498 | -0.06081 | 0.008277 | 0.001819 | 53.98505 |
| 2.48E-13 | rs1678717 | ENSG00000112164 | G | A | 29623 | 0.566444 | -0.06063 | 0.008283 | 0.001805 | 53.57585 |
| 3.45E-13 | rs1678701 | ENSG00000112164 | A | G | 29498 | 0.42352 | -0.06056 | 0.008325 | 0.001791 | 52.92495 |
| 4.41E-13 | rs9380795 | ENSG00000112164 | A | G | 29623 | 0.569676 | -0.06004 | 0.00829 | 0.001767 | 52.44447 |
| 6.07E-13 | rs2235867 | ENSG00000112164 | A | G | 29290 | 0.875837 | 0.090118 | 0.012518 | 0.001766 | 51.8235 |
| 7.07E-13 | rs9380787 | ENSG00000112164 | G | T | 29616 | 0.557233 | -0.05932 | 0.008265 | 0.001737 | 51.51733 |
| 7.22E-13 | rs6458073 | ENSG00000112164 | T | A | 29623 | 0.422714 | -0.05962 | 0.008309 | 0.001735 | 51.47715 |
| 7.61E-13 | rs1738199 | ENSG00000112164 | A | G | 29505 | 0.56506 | -0.05947 | 0.008297 | 0.001738 | 51.37531 |
| 1.06E-12 | rs9394550 | ENSG00000112164 | A | G | 29622 | 0.568908 | -0.05903 | 0.008289 | 0.001709 | 50.71804 |
| 1.61E-12 | rs6931736 | ENSG00000112164 | A | G | 29508 | 0.571695 | -0.05872 | 0.008312 | 0.001688 | 49.90378 |
| 6.26E-12 | rs4714193 | ENSG00000112164 | T | G | 29497 | 0.569479 | -0.05711 | 0.008308 | 0.001599 | 47.24317 |
| 6.45E-12 | rs1678682 | ENSG00000112164 | G | A | 29508 | 0.565619 | -0.057 | 0.008298 | 0.001597 | 47.18271 |
| 7.73E-12 | rs1738241 | ENSG00000112164 | T | C | 29623 | 0.56028 | -0.0566 | 0.008271 | 0.001578 | 46.82896 |
| 1.95E-10 | rs10305420 | ENSG00000112164 | T | C | 29085 | 0.364332 | 0.054806 | 0.00861 | 0.001391 | 40.51935 |
| 2.07E-10 | rs114977861 | ENSG00000112164 | C | T | 3243 | 0.012951 | 0.693724 | 0.109145 | 0.012304 | 40.37382 |
| 1.25E-08 | rs9380816 | ENSG00000112164 | C | T | 29623 | 0.437784 | 0.04712 | 0.008277 | 0.001093 | 32.41034 |
| 2.53E-08 | rs9349107 | ENSG00000112164 | A | G | 29507 | 0.441758 | 0.046156 | 0.008285 | 0.001051 | 31.03394 |
| 2.96E-08 | rs9357297 | ENSG00000112164 | C | T | 29496 | 0.330319 | 0.048504 | 0.008749 | 0.001041 | 30.73053 |
| 4.77E-08 | rs72857945 | ENSG00000112164 | G | A | 29508 | 0.329962 | 0.047774 | 0.00875 | 0.001009 | 29.8074 |
| 4.88E-08 | rs12213726 | ENSG00000112164 | T | C | 29623 | 0.328066 | 0.047717 | 0.008746 | 0.001004 | 29.76484 |
| 4.96E-08 | rs2281342 | ENSG00000112164 | C | T | 29502 | 0.327129 | 0.047821 | 0.00877 | 0.001007 | 29.72883 |

SNP, Single nucleotide polymorphism.

**Table S4** Multivariable Mendelian randomization estimates for the associations of GLP-1RAs, BMI, and T2DM with arrhythmias.

| **Outcome** | **Exposure** | **Method** | **OR (95% CI)** | **p-value** |
| --- | --- | --- | --- | --- |
| Atrial fibrillation | GLP-1RAs | IVW | 1.06 (0.60–1.89) | 0.848 |
|  | BMI | IVW | 1.00 (0.98–1.02) | 0.948 |
|  | T2DM | IVW | 1.06 (0.67–1.68) | 0.812 |
| Cardiac arrest and ventricular fibrillation | GLP-1RAs | IVW | 1.28 (0.36–4.52) | 0.705 |
|  | BMI | IVW | 1.01 (0.97–1.07) | 0.563 |
|  | T2DM | IVW | 1.19 (0.44–3.22) | 0.727 |

GLP1-RAs, glucagon-like peptide-1 receptor agonists; BMI, body mass index; T2DM, type 2 diabetes mellitus; CI, confidence interval; OR, odds ratio, IVW, inverse variance weighted.
